# Chimeric bacteriocin S5-PmnH engineered by domain swapping efficiently controls *Pseudomonas aeruginosa* infection in murine keratitis and lung models

**DOI:** 10.1038/s41598-022-09865-8

**Published:** 2022-04-19

**Authors:** Šarūnas Paškevičius, Viktorija Dapkutė, Audrius Misiūnas, Modestas Balzaris, Pia Thommes, Abdul Sattar, Yuri Gleba, Aušra Ražanskienė

**Affiliations:** 1 Nomads UAB, Geležinio vilko 29A, 01112 Vilnius, Lithuania; 2 Institute of Biotechnology, Vilnius University, Saulėtekio al. 7, 10257 Vilnius, Lithuania; 3 Evotec (UK) Ltd., Block 23, Alderley Park, Macclesfield, SK10 4TG Cheshire UK; 4 Nomad Bioscience GmbH, Biozentrum Halle, Weinbergweg 22, 06120 Halle (Saale), Germany

**Keywords:** Biotechnology, Drug discovery, Microbiology, Molecular biology

## Abstract

Rampant rise of multidrug resistant strains among Gram-negative bacteria has necessitated investigation of alternative antimicrobial agents with novel modes of action including antimicrobial proteins such as bacteriocins. The main hurdle in the clinical development of bacteriocin biologics is their narrow specificity and limited strain activity spectrum. Genome mining of bacteria for broadly active bacteriocins have identified a number of promising candidates but attempts to improve these natural multidomain proteins further, for example by combining domains of different origin, have so far met with limited success. We have found that domain swapping of *Pseudomonas* bacteriocins of porin type, when carried out between phylogenetically related molecules with similar mechanism of activity, allows the generation of highly active molecules with broader spectrum of activity, for example by abolishing strain resistance due to the presence of immunity proteins. The most broadly active chimera engineered in this study, S5-PmnH, exhibits excellent control of *Pseudomonas aeruginosa* infection in validated murine keratitis and lung infection models.

## Introduction

*Pseudomonas aeruginosa* (*P. aeruginosa*) are opportunistic Gram-negative pathogenic bacteria, causing both acute and chronic infections^1^. They harbor in their genome a large arsenal of virulence factors and antibiotic resistance determinants, conferring remarkable metabolic flexibility and the ability to adapt to multiple conditions, including the host immune response^1^. Furthermore, rapid development of resistance to previously effective antimicrobials, such as fluoroquinolones, aminoglycosides, and polymyxins, has been observed^2^. Biofilm-mediated resistance observed in chronic infections is another concern as the microbial cells grown in biofilms are less sensitive to antimicrobial agents and host immune response than the cells grown in free aqueous suspension^3^. The involvement of *P. aeruginosa* in a wide range of biofilm-related infections often leads to treatment failures^4^. Thus, new antimicrobial substances are urgently needed.

The possibility to use bacteriocins as new generation antimicrobials has been suggested previously^5^. *P. aeruginosa* produce colicin-type bacteriocins, pyocins, belonging to different classes: deoxyribonucleases, ribonucleases, pore-forming proteins, peptidoglycan synthesis-blocking proteins, lectin-like proteins, and bacteriophage tail-like protein complexes^6,7^. Several studies have described the use of natural pyocins for successful treatment of *P. aeruginosa* infections in various animal models^8–13^.

The main hurdles in the clinical development of bacteriocin antimicrobials are the proteinaceous nature of bacteriocins and their limited activity spectrum^7^. Being proteins, colicin type bacteriocins are expected to present the same challenges observed in the clinical development of bacteriophage lysins when used intravenously: short half-life, immunogenicity and weakened activity in serum^14,15^. These problems might be less relevant in the development of topical, oral or inhaled antimicrobials.

Previously, we successfully expressed in a plant transient expression system six different pyocins^12^. The only so far known *P. aeruginosa* pore-forming pyocin, S5, was found to be active against 40% of tested clinical isolates. S5 demonstrated superior activity compared to all other pyocins in reducing bacterial numbers in liquid culture and biofilm assays and also was most efficacious in protecting *G. mellonella* larvae from death due to *P. aeruginosa* infection^12^.

Pyocin S5 was first detected in PAO1 strain^16^ and has been shown to bind highly conserved ferripyochelin FptA receptor^17^. PyoS5 structure and functional domain analysis reveals that it is an elongated bacteriocin comprising a disordered region at its N terminus, two kTHB (kinked three-helix bundle) domains, and a C-terminal pore-forming domain. The N-terminal disordered region binds TonB1, kTHB domain 2 binds Common Polysacharide Antigen CPA, and kTHB domain 1 binds FptA^18^. PyoS5 delivers a pore-forming domain across the outer membrane to depolarize the cell^19^. Self-inhibition of S5-producing strains is prevented by co-expression of an immunity gene, located downstream of the bacteriocin gene, resulting in production of membrane-integrated immunity protein consisting of three transmembrane helices (TMHs)^20^. Previous studies have highlighted that the protective immunity proteins of pore-forming domains within colicins fall into two subgroups, although the functional significance of this is unclear. A-type immunity proteins have four transmembrane helices, while E1-type immunity proteins have three transmembrane helices^21^. Based on the predicted number of transmembrane helices of its immunity protein, the pore-forming domain of PyoS5 belongs to the E1 type^16,18^.

One of the means to broaden S5 spectrum of activity would be to modify its killing domain to avoid its recognition by the immunity protein. Other *Pseudomonas* species (in particular *P. fluorescens*) harbour in their genomes a variety of genes coding for pore forming bacteriocin-like proteins^7,22^. Through the mining of GenBank database, we retrieved several sequences of putative pore-forming bacteriocins from different *Pseudomonas* species and selected six colicin E1-like and colicin A-like proteins. We then used the cytotoxic pore forming domains of these bacteriocins to construct chimeric proteins fusing them to the receptor-binding and translocation domains of pyocin S5. After in vitro activity tests of the constructed chimeras, we selected the best performing chimeric protein S5-PmnH. We tested activity of S5-PmnH in different animal disease models, *P. aeruginosa* keratitis and lung colonization murine models, using topical and inhaled application of this chimeric bacteriocin.

## Results

### Identification of *Pseudomonas* putative pore-forming bacteriocin sequences

Putative pore-forming bacteriocins from genus *Pseudomonas* have been retrieved from NCBI by BLAST search using as query pore-forming domain of pyocin S5 (pfam01024). After the analysis of BLAST results, we selected six most divergent putative pore forming bacteriocins from different *Pseudomonas* species: Pflu095 (*P. fluorescens* WP_016979095), Pflu373 (WP_014717373 from strain A506), Pflu794 (WP_081041794 from strain ATCC 17400), Pflu618 (WP_034155618 from strain H16), Pput259 (WP_098964259 from *P. putida* strain FDAARGOS_376) and PmnH from *P. synxantha* strain BG33R. PmnH is the only one of selected proteins that has been published previously. It has unusual architecture as it harbors two cytotoxic domains, colicin-M like domain and pore-forming domain. So far, only activity of its pore-forming domain have been demonstrated^22^.

**Figure 1:**
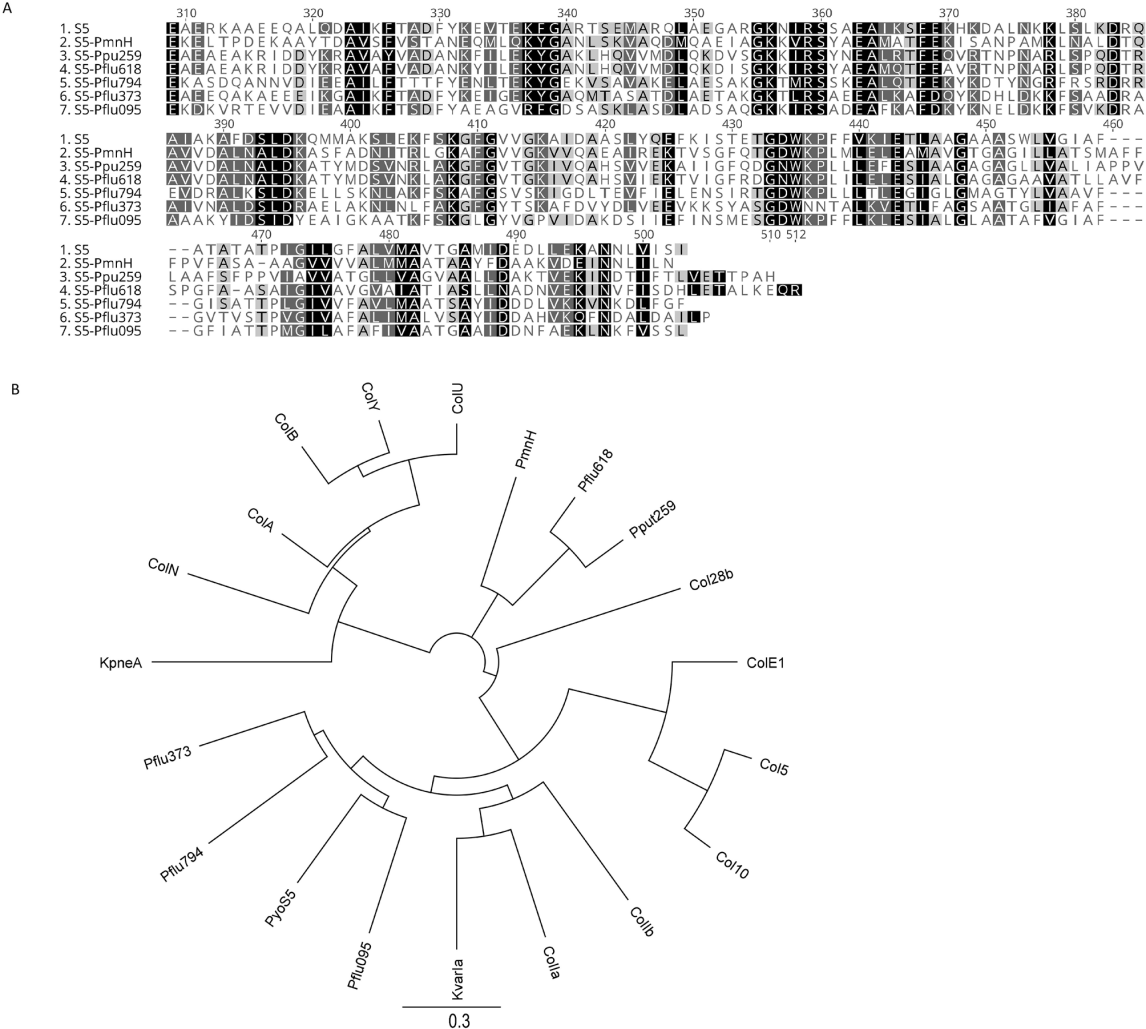
Sequence analysis of pore forming domains (pfam01024) of putative *Pseudomonas* bacteriocins. (**A**) ClustalW amino acid sequence alignment of *Pseudomonas* bacteriocins with pyocin S5. (**B**) Neighbor-Joining tree alignment of known *E. coli*, *Klebsiella* bacteriocins, pyocin S5, PmnH and putative *P. fluorescens and P. putida* bacteriocins. Col28B (CAA44310.1), ColE1 (AAA87379.1), Col10 (CAA57998.1), Col5 (CAA61102.1), ColIb (AAA23188.1), ColIa (WP_001283344.1), ColN (P08083.1), ColA (P04480.1), ColU (CAA72509.1), ColB (P05819.3), ColY (AAF82683.1), KpneA (SAV78255.1), KvarIa (KDL88409), Pyocin S5 (WP_003115311), PmnH (EIK72868.1), Pflu618 (WP_034155618), Ppu259 (WP_098964259), Pflu794 (WP_081041794), Pflu373 (WP_014717373), Pflu095 (WP_016979095).

Clustal W amino acid sequence alignment of pore-forming domains of six selected proteins showed 32–49% of identity with pyocin S5 (Fig. 1A). The amino acid sequences of pfam 01024 domains of bacteriocins were subjected to phylogenetic tree analysis along with pore-forming domains of some described pore-forming colicins, klebicins and pyocin S5 (Fig. 1B). Two major phylogenetic groups could be distinguished: pore forming domains of Pflu794, Pflu373 and Pflu095 are most related to pyocin S5 and belong to the group of E1-type proteins, and Pflu618 and Put259 are most related to PmnH and belong to A-type protein group (Fig. 1B).

### Construction and plant expression of chimeric pyocins

Pore-forming domains of six selected *Pseudomonas* putative porins were used for the construction of the chimeric proteins. All chimeric proteins contain identical N-terminal end of first 309 a.a. of pyocin S5 including translocation, FptA binding and CPA binding domains^18^. The S5 fragment was fused to the cytotoxic domain of the putative pore-forming bacteriocins.

**Figure 2:**
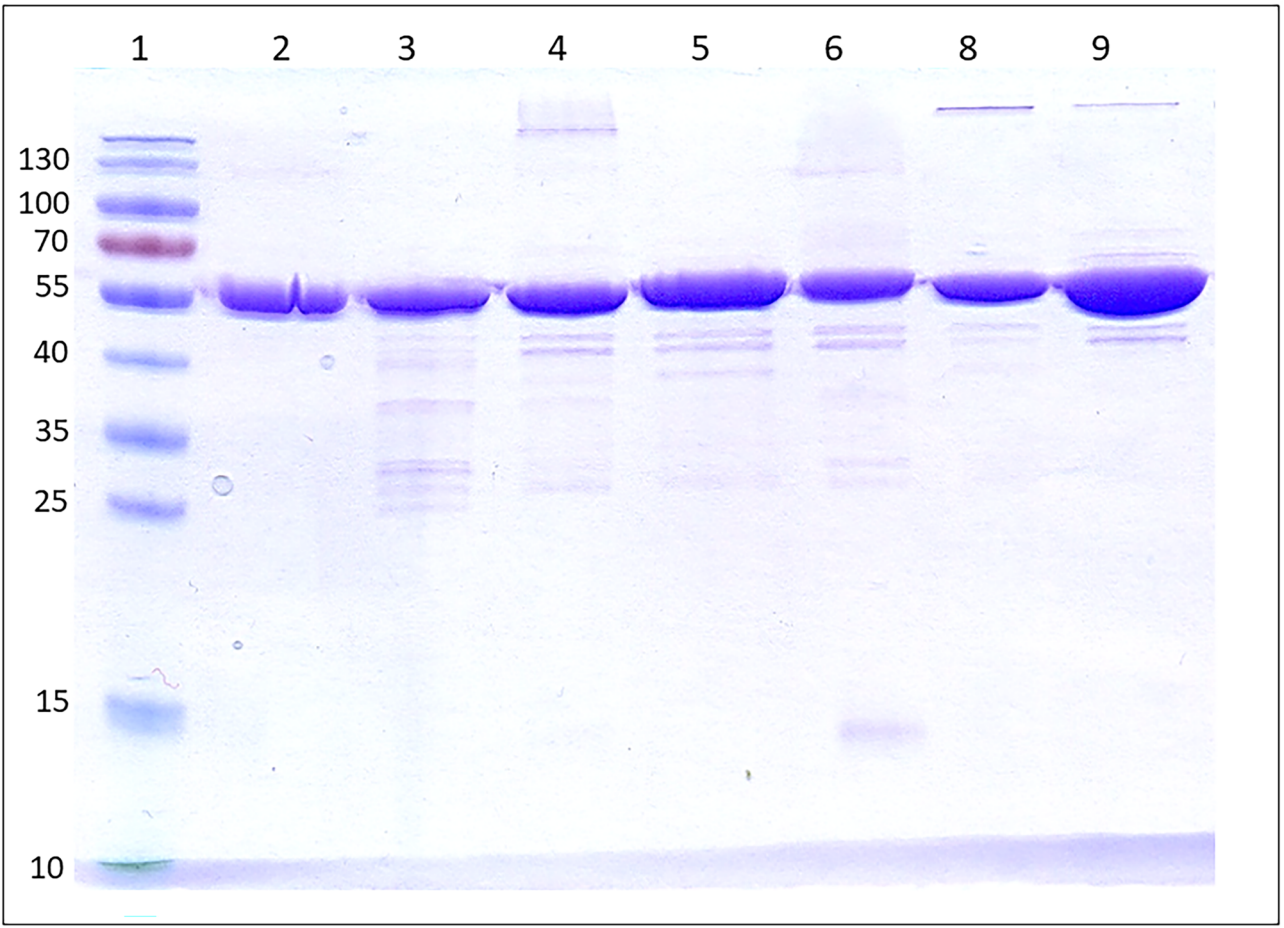
SDS-PAGE Coomassie staining of purified chimeric pyocins. Lane 1—PageRuler Prestained protein ladder (Thermo Fisher Scientific), lane 2—S5 (0.9 mg/ml), lane 3—S5-PmnH (0.9 mg/ml), lane 4—S5-Pflu095 (0.9 mg/ml), lane 5—S5-Pflu373 (0.9 mg/ml), lane 6—S5-Pflu794 (0.9 mg/ml), lane 8—S5-Pflu618 (0.9 mg/ml), lane 9—S5-Pput259 (1.4 mg/ml). 4 μl of proteins per lane.

All six chimeric proteins were successfully expressed in *Nicotiana benthamiana* transient expression system and purified by two-step chromatography (Fig. 2, Supplementary Text S1).

### In vitro activity of chimeric pyocins

A panel of 25 *P. aeruginosa* strains (from culture collections and clinical isolates, Supplementary Table S1) were subjected to genomic DNA extraction and PCR analysis using pyocin S5, pyocin S5 immunity protein and FptA receptor-coding gene specific primers. All 25 strains tested positive for the presence of *fptA*. Six strains (PA14, PAO1, HP6, HP7, ATCC 1960 and NCTC 13921) tested positive for amplification of both pyocin S5 and pyocin S5 immunity protein coding genes (Supplementary Fig. S1).

**Figure 3:**
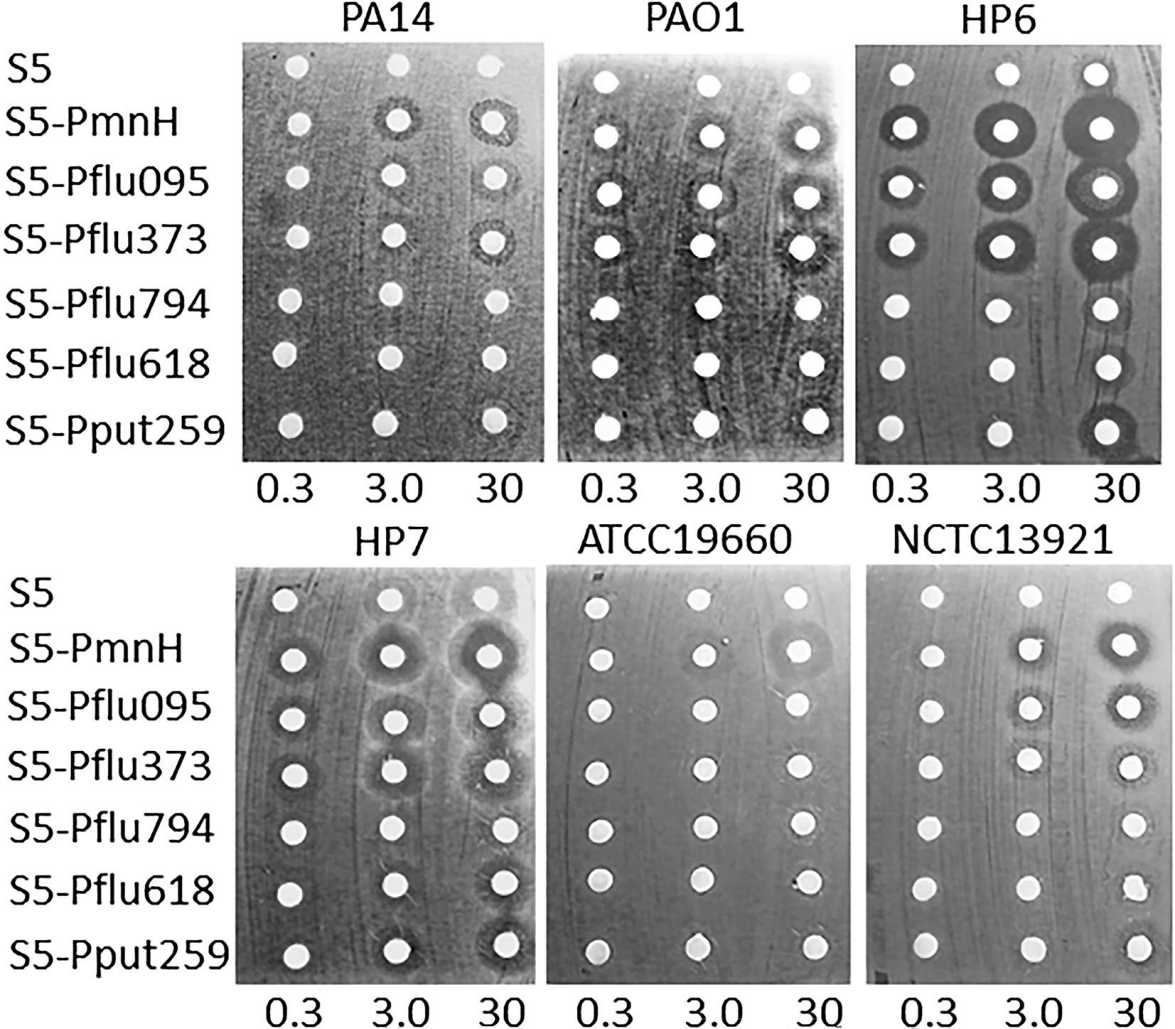
Pyocin S5 and chimeric pyocin activities on pyocin S5-producing *P. aeruginosa strains.* 0.3, 3 and 30 μg of S5 and chimeric bacteriocins were spotted on 6 mm Whatman discs placed on CAA agar lawns of different *P. aeruginosa* strains and incubated overnight.

Next, all 25 *P. aeruginosa* strains were subjected to agar disc-diffusion assay by spotting different amounts of purified pyocin S5 (0.3 μg, 3 μg and 30 μg) on bacteria lawns. Six *P. aeruginosa* strains were resistant to pyocin S5. Four of these six strains were pyocin S5 and immunity protein-encoding strains—PA14, PAO1, ATCC 1960 and NCTC 13921. Two cystic fibrosis isolates, 12-35708 and 12-29165 were also completely resistant to pyocin S5, despite the absence of S5, or S5 immunity protein coding genes. Surprisingly, pyocin S5 still had week inhibition effect on the lawns of two remaining S5 producer strains, clinical isolates HP6 and HP7. Turbid inhibition zones were detected when 3 μg and 30 μg of S5 were spotted on the lawn of these strains (Table 1, Fig. 3).

**Table 1:** Activity of chimeric pyocins on panel of *P. aeruginosa* strains as determined in agar disc-diffusion assay. *Activity detected only with 30 μg of protein, **activity detected with 3 μg of protein, ***activity detected with 0.3 μg of protein. Pyocin S5 killing and immunity protein genes-containing strains are in bold.

	Colicin E1-like				Colicin A-like		
	S5	S5-Pflu095	S5-Pflu373	S5-Pflu794	S5-PmnH	S5-Pflu618	S5-Pput259
Boston	***	***	***	*	***	**	***
**PA14**	**–**	**	**	**–**	***	**–**	**–**
**PAO1**	**–**	***	***	**–**	***	**	**
Bu002	***	***	***	**–**	***	**	**
A19	***	***	***	***	***	***	***
Pr335	***	***	***	**	***	**	***
EY76	***	***	***	**–**	***	**–**	**–**
BL77	***	**	**	**–**	**	*	*
UR78	***	***	***	**–**	***	*	*
BR79	***	**	**	**–**	**	**–**	*
BI80	***	***	***	**–**	***	*	*
HP1	***	***	***	**–**	***	**	***
**HP6**	*	***	***	*	***	*	**
**HP7**	**	***	***	**–**	***	**	**
HP40	***	***	***	*	***	**	**
HP41	***	***	***	**–**	***	**	**
HP52	***	**	**	**–**	**	*	*
HP75	**	*	**	**–**	**	**–**	**–**
ATCC 29260	***	***	***	**–**	***	*	*
12-35708	**–**	**–**	**–**	**–**	**–**	**–**	**–**
13-18499	***	**	**	**–**	**	*	*
12-29165	**–**	**–**	**–**	**–**		**–**	**–**
**ATCC 19660**	**–**	*	**–**	**–**	**	**–**	*
NCTC 13437	**	*	*	**–**	*	**–**	**–**
**NCTC 13921**	**–**	***	**	*	***	*	*

As the next step we analyzed the activity of S5 chimeras in agar disc diffusion assay. Two chimeric proteins, S5-Pflu095 and S5-PmnH demonstrated broadened activity spectrum in comparison to pyocin S5. Both chimeras formed inhibition zones on the lawns of all six S5-producing strains. The inhibition zones on HP6 and HP7 lawns were significantly larger and clearer than those formed by S5. These chimeric proteins also demonstrated activity similar to S5, with only small variations, on all other remaining *P. aeruginosa* strains (Table 1, Fig. 3). Between the two proteins, S5-PmnH demonstrated slightly superior activity and was selected for further experiments.

Chimeric bacteriocin S5-Pflu373 also demonstrated good activity profile, similar to S5-Pflu095, with exception that it was not active on one of S5 producers, ATCC 19660. S5-Pflu628 and S5-Pput259 chimeras performed less well and had weaker and less broad activity in comparison to pyocin S5, while S5-Pflu794 demonstrated only week activity on 6 tested strains (Table 1, Fig. 3). None of the chimeric proteins inhibited growth of two cystic fibrosis isolates, 12-35708 and 12-29165.

### Efficacy of S5-PmnH in *P. aeruginosa* keratitis models of disease

Two types of *P. aeruginosa* strains can be isolated from keratitis cases, cytotoxic *P. aeruginosa* strains are mainly causing keratitis related to contact-lens wear, while invasive strains are mostly causing disease in post-surgical complications^23^. We aimed to investigate if both type of strains could be targeted by S5-PmnH in disease models. Cytotoxic and invasive strains can be distinguished by genotyping the effector protein coding genes of their type III secretion systems (TTSS). Invasive strains were found to possess both *exoS* and *exoT* genes whereas cytotoxic strains appeared to have lost *exoS* but presented *exoT* and *exoU* genes^24–26^. We selected for our experiments cytotoxic strain ATCC 19660 (*exoT*, *exoU*) and invasive strain PAO1 (*ExoY*, exoT, exoS)^27^. Both strains are pyocin S5 producers and immune to pyocin S5, but both are sensitive to S5-PmnH.

### S5-PmnH treatment can reduce *P. aeruginosa* bacterial numbers in porcine corneas ex vivo

We first investigated the possibility to use S5-PmnH for eradication of *P. aeruginosa* colonizing the cornea in an ex vivo model, the dissected porcine corneas. Porcine corneas were colonized with invasive strain PAO1 or cytotoxic strain ATCC 19660. S5-PmnH MIC determined by agar dilution method against PAO1 was 4 µg/ml (0.07 nmol/ml) and against ATCC 19660 was 32 µg/ml (0.57 nmol/ml).

**Figure 4:**
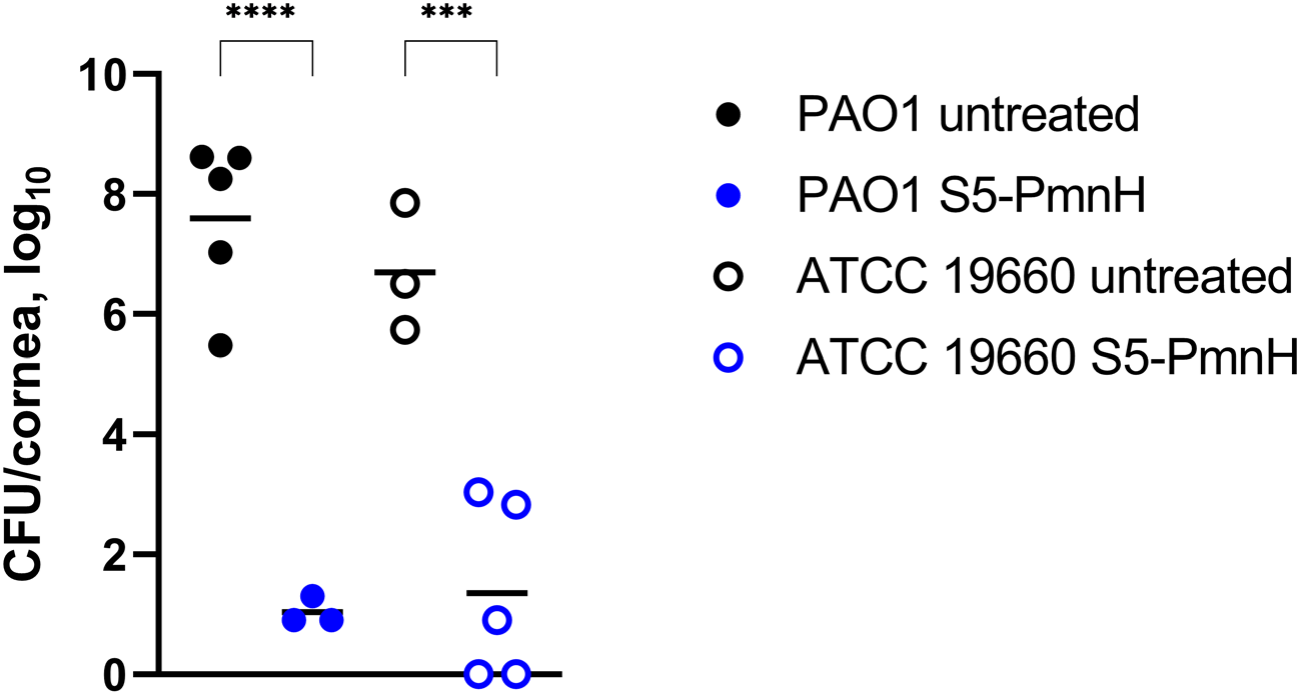
CFU counts in ex vivo porcine corneas, infected with *P. aeruginosa* ATCC 19660 or PAO1 and treated with S5-PmnH. 3 × 10^4^ CFU of *P. aeruginosa* ATCC 19660 strain or 0.4 × 10^4^ CFU of *P. aeruginosa* PAO1 strain were applied to cornea and incubated for 16–20 h at 37 °C. Then 5 µg of S5-PmnH were applied to cornea and incubated for additional 16–20 h. Statistical significances of the quantitative data were analyzed using GraphPad Prism software by the 1-way repeated measures ANOVA and Bonferroni’s’ correction for multiple comparisons. Mean is indicated by horizontal bar. ***P ≤ 0.001, ****P ≤ 0.0001 vs vehicle-treated mice.

In order to obtain *P. aeruginosa* colonization, porcine corneas were incubated with 3 × 10^4^ CFU of *P. aeruginosa* ATCC 19660 or 0.4 × 10^4^ CFU of *P. aeruginosa* PAO1 for 16 to 20 h. Then 5 µg of S5-PmnH were applied to the cornea and incubated for additional 16–20 h. At the end of the experiment PAO1 burden in untreated corneas reached an average of 7.6 log_10_ CFU/cornea, while S5-PmnH-treated corneas the burden was only an average of 10 CFU per cornea, demonstrating 6.6 log_10_ reduction. In ATCC 19660 colonized corneas S5-PmnH treatment reduced CFU number by 5.3 log_10_ (Fig. 4). Thus, in ex vivo porcine corneas, S5-PmnH efficiently reduced *P. aeruginosa* colonization by both strains.

### S5-PmnH efficiently kills bacteria and prevents acute disease in murine keratitis models

#### Infection by cytotoxic strain ATCC 19660

**Figure 5:**
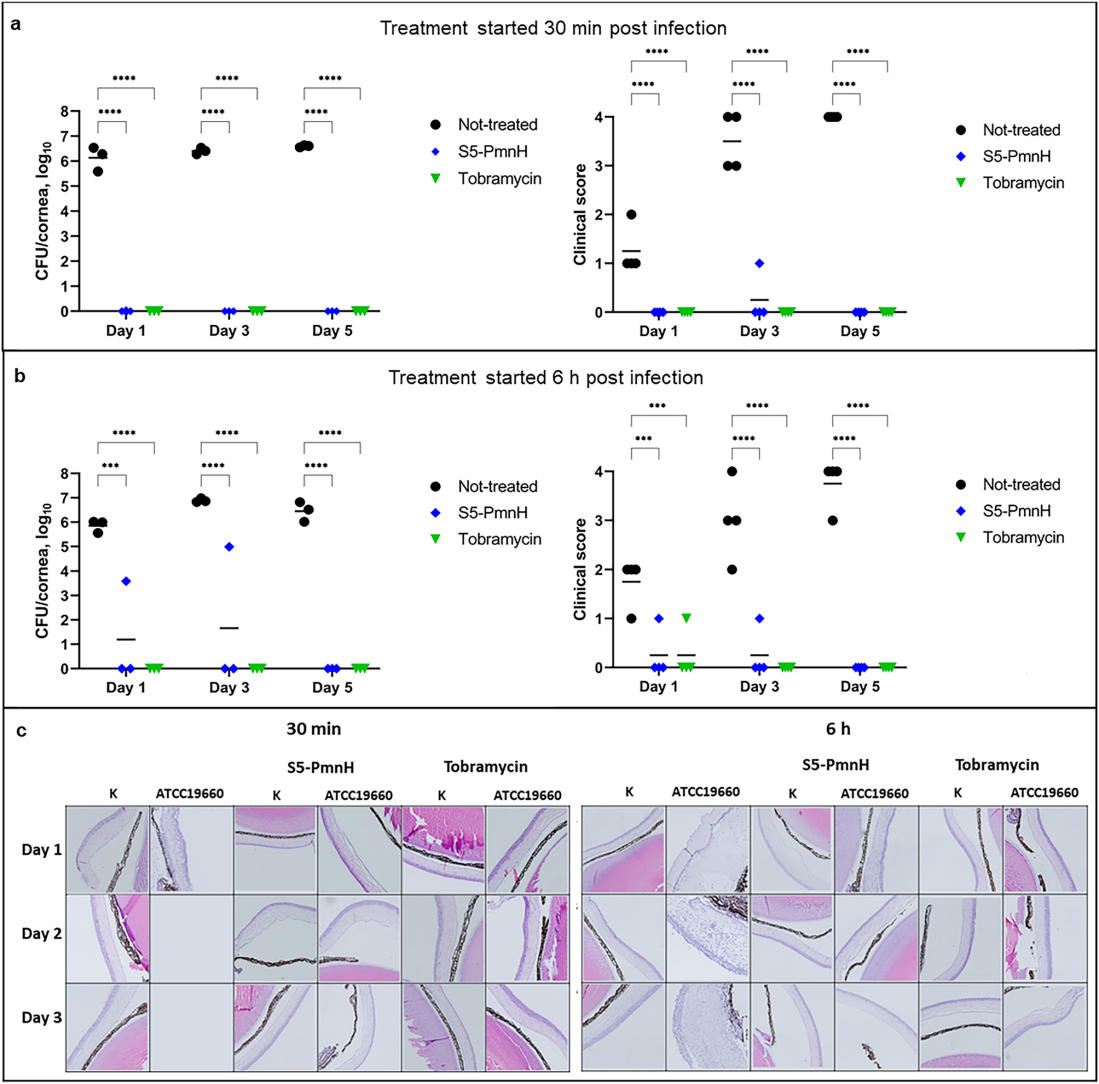
Mice cornea infection by cytotoxic strain ATCC 19660 and treatment by S5-PmnH or tobramycin. (**a**) CFU counts and clinical scores of mice eyes, when treatment started 30 min post-infection. (**b**) CFU counts and clinical scores of mice eyes, when treatment started 6 h post infection. (**c**) Hematoxylin–eosin staining of cornea sections. Left panel—treatment with pyocin started 30 min post-infection. Uninfected eyes—no observed microscopic aberations. Infected control eyes: day 1—strong corneal inflamation, days 3 and 5—the histology was not possible because of disrupted structure of the eye. Weak edema in the corneal stroma is observed in all infected eyes treated with S5-PmnH and with tobramycin. Right panel—treatment with pyocin started 6 h post infection**.** Uninfected eyes: no marked cornea abberations. Infected untreated eyes: day 1—thinning of corneal epithelium, thickening of stroma, acute inflammation, days 3 and 5—acute suppurated inflammation of cornea. S5-PmnH-treated infected eyes: day 1—acute inflammation of cornea, days 3 and 5—no marked aberrations, week edema of corneal stroma. Tobramycin-treated infected eyes: day 3 and 5—slight thickening of epithelium. Statistical significances of the quantitative data were analyzed using GraphPad Prism software by the 2-way repeated measures ANOVA and Dunnett’s correction for multiple comparisons. Mean is indicated by horizontal bar. ***P ≤ 0.001, ****P ≤ 0.0001 vs vehicle-treated mice.

For induction of keratitis the mice were anaesthetized, the cornea of left eye was scratched with a sterile needle and *P. aeruginosa* ATCC 19660 (4 × 10^6^ CFU) was applied to the corneal surface. The treatment by S5-PmnH, tobramycin or PBS (mock-treatment) was started 30 min post infection or 6 h post infection. When the treatment was started 30 min after the infection, no viable *P. aeruginosa* were isolated from infected eyes in both S5-PmnH and tobramycin-treated groups on 1, 3 or 5 dpi. By contrast, the bacterial burden in infected and untreated eyes reached 6–7 log_10_ CFU/cornea (Fig. 5a, left panel).

The visual inspection by microscope of infected untreated eyes revealed acute disease signs: slight to dense opacity of cornea at 1 dpi, and dense opacity and sometimes cornea perforation at 3 dpi. At 5 dpi all the mice had cornea perforations. No signs of disease were observed in majority of samples treated by S5-PmnH or tobramycin (Fig. 5a, right panel, Supplementary Fig. S2). The histopathology examination of infected and mock-treated eyes revealed strong corneal inflammation at 1 dpi, and disrupted structure of the eye at 3 dpi and 5 dpi. Only weak oedema in the corneal stroma was observed in all infected eyes treated with PyoS5-PmnH and with tobramycin (Fig. 5c). Thus, both S5-PmnH and tobramycin completely eradicated *P. aeruginosa* ATCC 19660 and prevented the disease when the treatment was started almost immediately, 30 min, after infection.

We repeated the experiment with delayed treatment time, allowing the infection to establish for 6 h. Similar to the previous experiment, the average CFU burden in infected mock-treated eyes reached 6.1 to 6.6 log_10_ CFU/cornea. In the tobramycin-treated group of mice, no viable bacteria were isolated on 1, 3 or 5 dpi. In the S5-PmnH-treated group viable bacteria were isolated from one mouse at 1 dpi (3.6 log_10_ CFU/cornea) and from one mouse at 3 dpi (5 log_10_ CFU/cornea). No viable bacteria were isolated from neither of three mice at 5 dpi (Fig. 5b, left panel). The clinical score evaluation of infected and mock-treated eyes revealed very similar picture to the previous experiment: the opacity of cornea started at 1 dpi and 3 out of 4 mice had cornea perforations at 5 dpi. Only mild disease signs were observed in two S5-PmnH-treated and one tobramycin-treated mice at 1 and 3 dpi and all corneas were completely clear at 5 dpi (Fig. 5b, right panel, Supplementary Fig. S3). The histopathology examination of mock-treated eyes revealed the thinning of corneal epithelium, thickening of stroma, acute inflammation at 1 dpi and acute suppurated inflammation of cornea at 3 dpi and 5 dpi. PyoS5-PmnH-treated infected eyes at 1 dpi presented signs of acute inflammation of cornea and no marked aberrations at 3 and 5 dpi, just weak oedema of corneal stroma was observed. Tobramycin-treated infected eyes at 3 and 5 dpi showed slight thickening of epithelium (Fig. 5c, right panel). Thus, here again S5-PmnH treatment was efficient in eradicating *P. aeruginosa* from infected corneas and in preventing the establishment and progress of disease.

#### Infection by invasive strain PAO1

**Figure 6:**
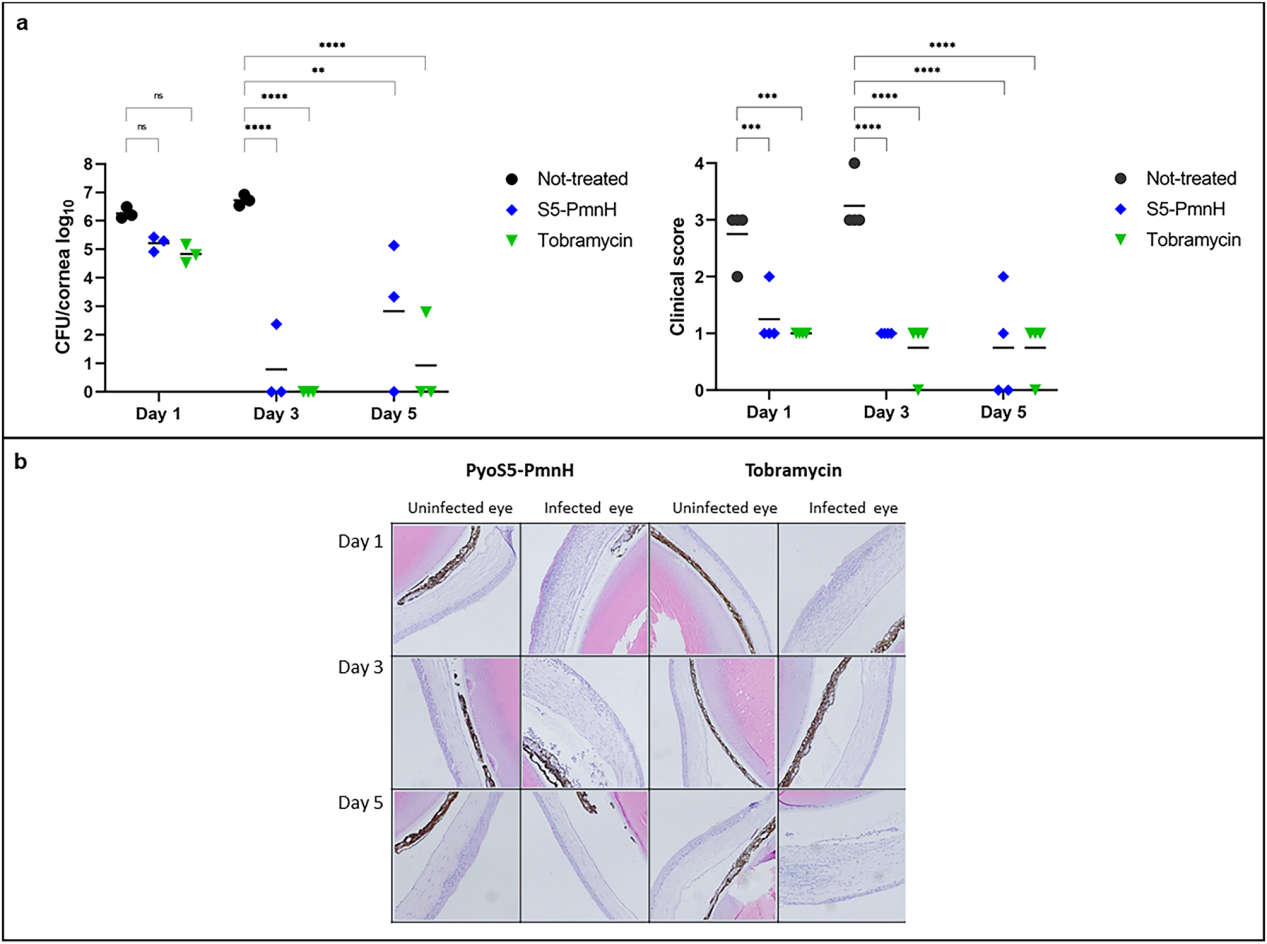
Mice cornea infection by invasive strain PAO1 and treatment by S5-PmnH or tobramycin. The corneas of left eye of mice were infected with 4 × 10^6^ CFU of *P. aeruginosa* PAO1 strain. The treatment with 20 µg of S5-PmnH or 140 µg of tobramycin started 6 h post-infection and was applied twice daily. (**a**) CFU counts in mice corneas and cornea clinical scores at 1, 3 and 5 dpi. (**b**) Hematoxylin–eosin staining of cornea sections. Uninfected eyes: no marked aberrations, weak edema in corneal stroma observed in most samples. Infected S5-PmnH-treated eyes: days 1 and 3—acute inflammation of cornea, day 5—no aberrations. Infected tobramycin-treated eyes: Day 1—acute inflammation of cornea, local cornea lesions, Day 3—thinning of cornea epithelium, strong edema of the corneal stroma; day 5—local inflammation of cornea, thinning and degeneration of cornea epithelium. Statistical significances of the quantitative data were analyzed using GraphPad Prism software by the 2-way repeated measures ANOVA and Dunnett’s correction for multiple comparisons. Mean is indicated by horizontal bar. **P ≤ 0.01, ***P ≤ 0.001, ****P ≤ 0.0001 vs vehicle-treated mice. Not significant (ns) P > 0.05.

Next, we examined the efficacy of S5-PmnH for treatment of cornea infection by invasive *P. aeruginosa* PAO1. The infection and treatment procedures were similar to the previous experiment and treatment was started 6 h post infection. The control group of infected mice suffered from severe disease and were euthanized at 3 dpi. At 1 dpi, bacterial burden in mock-treated group of mice reached 6.26 log_10_ CFU/cornea. The S5-PmnH and tobramycin treatment reduced burden by average 1.04 log_10_ CFU/cornea and 1.42 log_10_ CFU/cornea, respectively. At 3 dpi no viable bacteria were isolated from all three tobramycin-treated mice, and from two S5-PmnH-treated mice. The cornea from the third S5-PmnH-treated mouse contained 2.38 log_10_ CFU of viable bacteria. By contrast, burden in the control group of mice reached 6.73 log_10_ CFU/cornea. At 5 dpi, no bacteria were isolated from two tobramycin-treated and one S5-PmnH-treated mice. One mouse from the tobramycin treated group contained 2.8 log_10_ CFU/cornea. The two remaining mice from the S5-PmnH treated group contained 5.14 log_10_ and 3.34 log_10_ CFU/cornea (Fig. 6a, left panel).

Clinical examination revealed stronger disease symptoms compared to the cytotoxic strain ATCC 19660. Mock-treated eyes presented severe disease signs at 1 dpi (clinical score 2–3). Several S5-PmnH-treated and tobramycin-treated eyes presented mild clinical scores (1–2) starting from 1 dpi to the end of experiment (Fig. 6a, right panel, Supplementary Fig. S3).

Histological examination of uninfected eyes revealed no marked aberrations, although weak edema in corneal stroma was observed in most samples. Infected S5-PmnH-treated eyes presented signs of acute inflammation of cornea at 1 dpi and 3 dpi. Infected tobramycin-treated eyes presented signs of acute inflammation of cornea at 1 dpi, thinning of cornea epithelium and strong edema of the corneal stroma at 3 dpi and local inflammation of cornea, thinning and degeneration of cornea epithelium at day 5 (Fig. 6B).

In conclusion, S5-PmnH efficiently reduced bacterial burden and prevented acute disease regardless of whether a cytotoxic or invasive strain was used for infection. However, despite lower S5-PmnH MIC against *P. aeruginosa* PAO1 than against *P. aeruginosa* ATCC 19660, the chimeric pyocin more efficiently eradicated the cytotoxic strain of *P. aeruginosa* and prevented the establishment of disease; a similar effect was observed for tobramycin.

#### S5-PmnH efficiently eradicates lung colonization by *P. aeruginosa* in a murine model of disease

Mice were infected intranasally (IN) with *P. aeruginosa* ATCC 27853 strain. One hour later, 2.5, 25 and 250 µg/mouse of S5-PmnH were administered IN once to both nares of the mouse. 5 h later mice were euthanized and lung burden of *P. aeruginosa* ATCC 27853 was evaluated.

**Figure 7:**
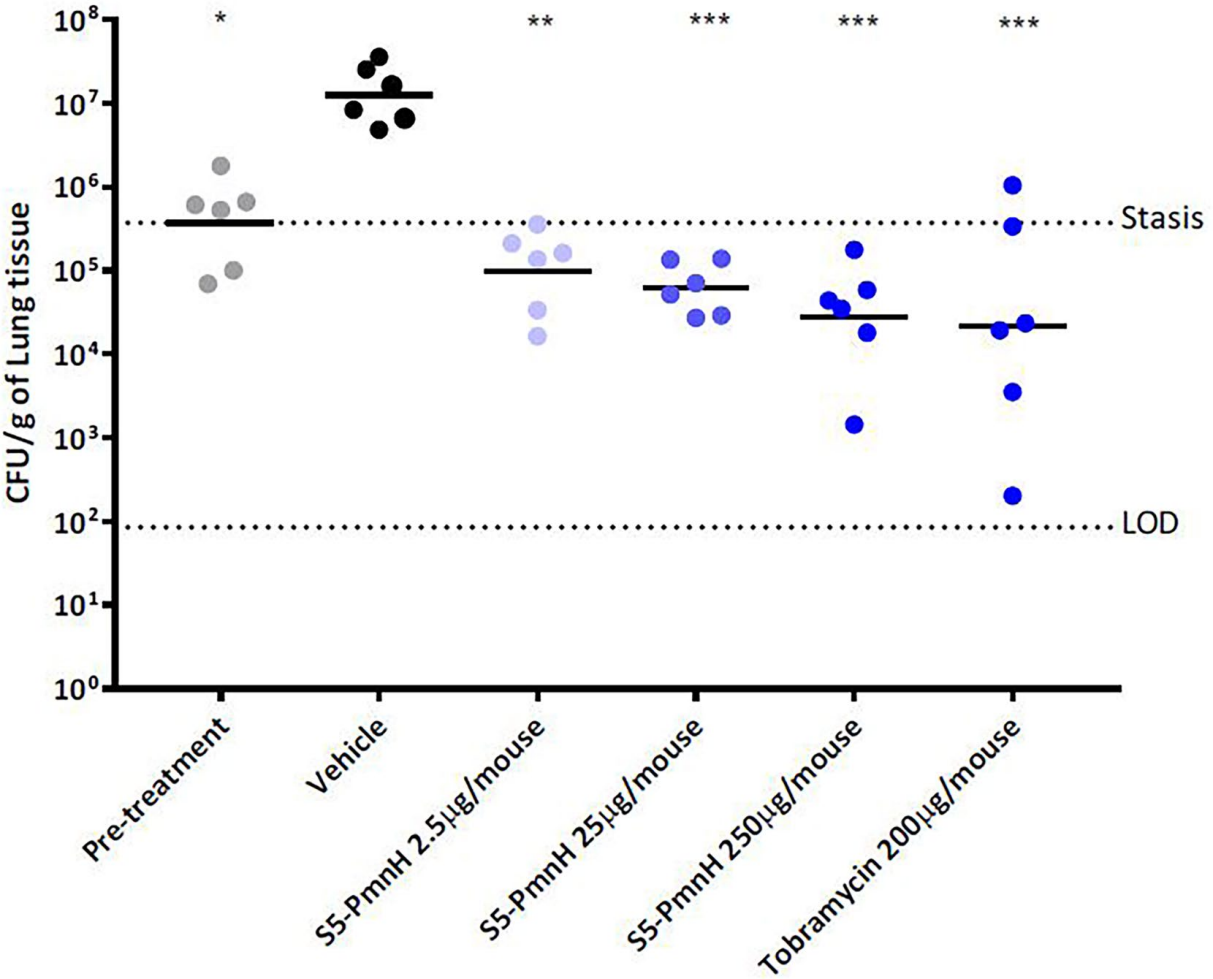
Scatterplot of terminal lung burden following IN infection with *P. aeruginosa* ATCC 27853. The data from the culture burdens were analyzed using appropriate non-parametric statistical models (Kruskal–Wallis using Conover-Inman to make all pairwise comparisons between groups) with StatsDirect (v. 3.3.3). The geometric mean burden of each treatment is indicated by the horizontal bar. *P ≤ 0.05, **P ≤ 0.0005, ***P ≤ 0.0001, compared to vehicle control. *LOD* limit of detection.

5 h post infection, *P. aeruginosa* ATCC 27853 burden in the mock-treated mice reached 1.24 × 10^7^ CFU/g of lung tissue, corresponding to an increase of 1.53 log_10_ CFU/g from 1 h post infection. S5-PmnH administered IN at 2.5 µg/mouse reduced lung burden by 2.1 log_10_ CFU/g, administered at 25 µg/mouse by 2.31 log_10_ CFU/g and administered at 250 µg/mouse by 2.66 log_10_ CFU/g. The bacterial burden was reduced to below the level of stasis (pre-treatment) in all S5-PmnH treatment groups: in 2.5 µg/mouse group by 0.58 log_10_ CFU/g, in 25 µg/mouse group by 0.78 log_10_ CFU/g and in 250 µg/mouse group by 1.13 log_10_ CFU/g. Increased reduction in burden was observed with higher dose levels of S5-PmnH, however the differences were not statistically significant. Tobramycin administered IN once at 200 µg/mouse reduced bacterial burden by 2.75 log_10_ CFU/g compared to vehicle, corresponding to 1.23 log_10_ CFU/g below the level of stasis. Higher variability was observed in this group compared to S5-PmnH or vehicle treatments (Fig. 7).

## Discussion

The attempts to use naturally occurring antibacterial proteins for fighting pathogenic bacteria started a while ago. Bacteriophage lysins (endolysins), peptidoglycan hydrolases, were the first ones to be explored. Bacteriophage lysins have been shown to effectively target numerous Gram-positive pathogens^28^, the most advanced of those, lysin PlySs2 (Exebacase), has already entered Phase III clinical trials. Similar to colicin-like bacteriocins, the modular structure of bacteriophage lysins provides an opportunity to engineer enzymes with altered bacteriolytic activity, and several active new hybrid molecules were constructed by swapping the domains of different lysins^29^. Fighting Gram-negative pathogens, however, is a much greater challenge as outer membrane of the Gram-negative pathogens prevents the access of the potentially antibacterial biologics to the periplasm or bacterial cell cytoplasm and nucleus. Several attempts have been made to enable endolysins to penetrate the outer membrane. For instance, endolysins have been engineered by adding polycathionic or/and hydrophobic/amphipathic peptides to make them able to cross outer membrane (Artilysins™)^30^. As an alternative approach, the attempts have been made to equip colicin-like bacteriocins with lytic domains of endolysins, thus enabling such engineered proteins to translocate to the periplasm of Gram-negative bacteria^31,32^. In first such attempt, the FyuA binding domain of pesticin, bacteriocin from *Yersinia pestis*, was fused to the N-terminus of T4 lysozyme. This hybrid toxin was shown to kill specific *Yersinia* and pathogenic *E. coli* strains and, importantly, it was able to evade the pesticin immunity protein (Pim) giving it a distinct advantage over pesticin^31^. In another study, *P. aeruginosa* bacteriocin pyocin S2 domains responsible for surface receptor binding and outer membrane translocation were fused to the GN4 lysin to generate the PyS2-GN4 ‘lysocin’. PyS2-GN4 induced peptidoglycan cleavage and log-fold killing of *P. aeruginosa*, efficiently disrupted biofilms, and protected mice from *P. aeruginosa* challenge in a bacteremia model^32^. However, practical use of this chimeric bacteriocin is limited because of the narrow activity spectrum as it only targets *P. aeruginosa* strains carrying ferripyoverdine receptor FpvAI. Elfarash et al. used the DNAse domain of pyocin S2 and fused it to N-terminal part of pyocin S5, containing receptor binding and translocation domains, obtaining an active chimeric protein. S5-S2b chimera targeted strains with FptA receptor and a strain known to be immune to pyocin S2 was, as expected, resistant to the hybrid pyocin containing the S2 killing domain^17^. These and other previous attempts to engineer new active molecules for control of Gram-negative bacteria were invariably relying on combining domains of phylogenetically and/or functionally unrelated proteins. We therefore decided to construct chimeric molecules by swapping functional domains of closely related bacterial species with the same mechanism of antibacterial activity, namely chimeras of porin-porin type.

The only known pore forming bacteriocin of *P. aeruginosa* was first detected in PAO1 strain^16^. Pyocin S5 binds highly conserved ferripyochelin FptA receptor^17^. The exact prevalence of clinical *P. aeruginosa* strains producing S5 is unknown, but it was demonstrated in the bacteriocin prevalence study of catheter *P. aeruginosa* isolates that about 25% of these strains contained pyocin S5 coding gene^33^. Thus, these 25% of catheter isolates should be in theory resistant to pyocin S5. We have tested pyocin S5 and S5 immunity protein gene presence in 25 *P. aeruginosa* isolates in our in-house collection and found very similar results, 6 strains (or 24%) contained pyocin S5 and immunity protein coding gene sequences. As expected, the presence of pyocin S5 coding gene in these strains correlated with the resistance to the antimicrobial activity of pyocin S5. In our previous study, we also found that pyocin S5 was active against 40% of tested *P. aeruginosa* clinical isolates^12^. Thus, we speculated that if the immunity of S5 producing strains could be overcome by using cytotoxic domain other than S5, the spectrum of the chimera would be significantly broadened, and it could be able to target more than half of strains.

Towards this goal, we constructed six chimeric S5 pyocins, in which the pore-forming domain of S5 was replaced by pore-forming domains of putative bacteriocins from *Pseudomonas* species other than *P*. *aeruginosa*. All six chimeric pyocins were successfully expressed in *N. benthamiana* transient expression system, which is our system of choice for expressing colicin-like bacteriocins^12,34^. Partially purified chimeric proteins were subjected to agar drop test assay on 25 *P. aeruginosa* strains, six between them being producers of S5 and insensitive or only slightly sensitive to this porin.

The chimeric proteins demonstrated variable activity profiles, with three of them standing out as more broadly active than the parental strain S5: two with bacteriocin-like porin fragments coded by *P. fluorescens* (S5-Pflu095 and, S5-Pflu373) and one with pore forming domain from *P. synxantha* bacteriocin (S5-PmnH)*.* The chimeric protein S5-PmnH was active on all six S5-producing strains and did show comparable activity to S5 on all remaining tested *P. aeruginosa* isolates. This chimeric protein was selected for further experiments in murine models for topical treatment of two unrelated models of disease caused by *P. aeruginosa*: keratitis model and lung infection model.

Bacterial keratitis is mostly affecting contact lens wearers, although several other risk factors, such as ocular surface diseases, ocular traumas or ocular surgery, are also not negligible. A 5-year review of cases at Dubai hospital revealed that in 37% of bacterial keratitis case the causative agent of disease is *P. aeruginosa,* although this frequency may vary in different geographical regions^35,36^. Untreated bacterial keratitis usually results in blindness. Two types of *P. aeruginosa* strains are found in eyes affected by keratitis: cytotoxic strains that mainly cause keratitis in contact-lens wearers, and invasive strains mostly causing disease in post-surgical complications^23^. We aimed to investigate if both types of strains can be targeted by S5-PmnH in mice keratitis models.

Chimeric pyocin S5-PmnH demonstrated efficacy in both cytotoxic and invasive *P. aeruginosa* models of eye infection. In cytotoxic keratitis model, the treatment completely eliminated all bacteria one day post infection, if treatment was started 30 min after infection. When treatment was delayed for 6 h, viable bacteria were isolated only from one mouse at 1 dpi and one mouse at 3 dpi. Visual inspection and histological examination of eyes of all treated mice in both experiments did not reveal any significant lesions, with no marked difference from non-infected eyes. By contrast, very strong keratitis symptoms were observed in all infected and untreated eyes at 3 and 5 dpi. Thus, S5-PmnH treatment efficiently eradicated *P. aeruginosa* from cornea infected by the cytotoxic strain *P. aeruginosa* ATCC 19660 and prevented the establishment and progress of the disease. A similar experiment was performed using the invasive *P. aeruginosa* strain PAO1 with treatment starting 6 h after infection. Although both treatments (S5-PmnH or tobramycin) were less efficient in eradicating bacteria, the progress of disease was greatly reduced in all treated mice. Taken together, these studies clearly demonstrate the efficacy of chimeric pyocin in this disease model, comparable to that of the standard of care antibiotic tobramycin. Further experiments are necessary to evaluate the efficacy of S5-PmnH against bacteria growing as biofilms and against bacteria which have been internalized by eukaryotic cells.

*Pseudomonas aeruginosa* is also a frequent cause of lung infections, including hospital-acquired pneumonia (HAP) and ventilator-associated pneumonia (VAP). This pathogen has a worsening global trend towards more likely displaying MDR phenotypes^37^. In addition, *P. aeruginosa* can cause chronic lung infections in patients with cystic fibrosis (CF) and non-CF bronchiectasis. Acquisition of *P. aeruginosa* is associated with increased morbidity and mortality in patients with CF, and is an important factor in the development and progression of CF respiratory disease^38–40^. Patients with CF are at very high risk of developing infections with multidrug-resistant (MDR) *P. aeruginosa*, owing to the frequent and often prolonged courses of oral, intravenous, and aerosolized antibiotics that are used to treat the chronic lung disease of CF^41^. In our validated mouse lung colonization model, one single application of chimeric pyocin S5-PmnH, even at the lowest concentration used, 2.5 μg, reduced bacterial burden in the lungs below the level of stasis. In a similar study, pyocin S5 had been shown successfully to eradicate lung colonizing *P. aeruginosa* in mice with superior activity compared to pyocins L1, S2 and AP41^11^. The results obtained between the two studies cannot be compared directly because of differences in experimental protocols and *P. aeruginosa* strains used. Our study has limitations, as we only performed short time bacteremia evaluation. However, the successful results obtained with chimeric pyocin S5-PmnH used in our study back up the possibility to use porin type bacteriocins for efficiently targeting bacteria in lungs and also demonstrate the endless possibilities of engineering bacteriocins with modified or broadened activity spectra.

## Methods

### Bacterial strains and cultures

Unless otherwise stated, *P. aeruginosa* strains were prepared by culturing in Lysogeny Broth (LB) medium (Roth) or Casamino Acids (0.5% Bacto™ Casamino acids, 5.2 mM K_2_HPO_4_, 5 mM MgSO_4_) medium (BD Bacto) at 37 °C under shaking conditions (200 rpm); overnight cultures were prepared by inoculation from frozen stocks.

*P. aeruginosa* strains used in experiments are described in Supplementary Table S1.

### Construction of chimeric pyocins

The open reading frames encoding for PmnH (*P. synxantha* EIK72868), Pflu095 (*P. fluorescens* WP_016979095), Pflu373 (*P. fluorescens* WP_014717373), Pflu794 (*P. fluorescens* WP_081041794), Pflu618 (*P. fluorescens* WP_034155618) and Pput259 (*P. putida* WP_098964259) optimized for expression in *N. benthamiana* were synthetized by Thermofisher Scientific (USA). Pyocin S5 synthesis and expression vector construction were described previously^12^.

Chimeric proteins were constructed as follows: N-terminal end of pyocin S5 (coding sequence of 1–310 aa containing receptor binding and translocation domains of this pyocin) was amplified with sequence-specific primers flanked by *Bsa*I recognition sites (Supplementary Table S2). Cytotoxic domains of all non-*P*. *aeruginosa* putative bacteriocins were amplified with sequence specific primers flanked with *Bsa*I recognition sites (Supplementary Table 2). Each killing domain fragment was paired with S5 fragment and both fragments were inserted in *Bsa*I digested pICH29912, assembled TMV-based MagnICON vector^42^. Obtained plasmids were used to transform *A. tumefaciens* GV3101. The datasets generated during the current study are available in supporting information. The sequences of chimeric pyocins are presented in Supplementary Text S2.

### Chimeric pyocins expression in plants

*Nicotiana benthamiana* plants were grown in a growth chamber at 25 °C with a 16 h light and 8 h dark photoperiod. Four-to-six-week-old plants were used for vacuum infiltration with recombinant *A. tumefaciens*. All plant experiments were performed in compliance to relevant institutional, national, and international guidelines and legislation.

*Agrobacterium* strains were inoculated from frozen stocks in 4 ml LB medium containing 50 µg/ml rifampicin and 50 µg/ml kanamycin and cultivated at 28 °C with shaking at 220 rpm. Overnight cultures were diluted 1:1000 starting from OD_595_ = 1.0 in tap water and supplemented with 0.05% Silwet L77 (Kurt Obermeier). *Agrobacterium* suspension was poured into a desiccator vessel, connected to a vacuum pump. The entire leaf system of a plant was then submerged into the suspension. Agroinfiltration was achieved by applying (till pressure of 200 mbar) and releasing vacuum through the pump. Plant leaves were harvested 5–6 days post agroinfiltration.

### Purification of plant-produced chimeric pyocins

Detailed protocols of purification are presented in Supplementary information (Supplementary Text S1). Pyocin S5 was purified as previously described by Ref.^12^.

### Agar disk-diffusion assay

Overnight *P. aeruginosa* cultures grown in CAA medium were equalized till OD_595_ = 1.0 in CAA and diluted 100×. Sterile cotton swab was briefly submerged in diluted microbial suspension, removing the excess of liquid by pressing it against the container wall. The swab was used for evenly streaking bacteria on plates containing growth medium with CAA solid agar (1.5%). 6 mm diameter sterile Whatman discs were placed on soft-agar and 0.3–30 μg of chimeric pyocins were spotted to paper disks. The plates were incubated overnight at 37 °C and bacteriocin inhibition zones were observed.

### Determination of MIC by agar dilution method

Aliquots of melted CAA (1.5% agar) medium (final volume—25 ml) pre-warmed to 51 °C were supplemented with 0.1 mg/ml BSA and with appropriate amounts of pyocin stock solutions of different concentrations (concentrations lowered by a factor of 2). Aliquots were poured into Petri plates. *P. aeruginosa* cultures were grown from single colony in CAA medium at 37 °C, 200 rpm until OD_595_ = 0.2 and diluted to 10^7^ CFU/ml in CAA medium. 1 µl of bacteria suspension were applied in three replicates on each test plate. Plates were left to incubate in 37 °C overnight. The determined MIC is a concentration of pyocin, where no confluent bacterial growth is observed.

### Ex vivo porcine corneas as model for bacterial keratitis

#### Preparation of porcine corneas

Porcine eyes were acquired from the nearest slaughterhouse. Enucleated eyes were stored at − 70 °C. Before the start of experiment, eyes were transferred to 4 °C for 1 h, then to room temperature for 1–2 h until the eyeballs were completely defrosted. Then eyeballs were individually placed in sterile plastic containers and submerged for 5 min in 2.5% Povidone-iodine (Betadine 100 mg/ml; EGIS Pharmaceuticals PLC), then twice washed with sterile PBS. The corneas were excised with sterile surgical blade No. #12. The excised corneas were stored in Minimum Essential Medium (MEM) supplemented with Non-Essential Amino Acids, l-Glutamine (2 mM), penicillin (200 U/ml) + spectinomycin (25 µg/ml) until further use at 4 °C up to two weeks.

#### Cornea infection and pyocin treatment

*P. aeruginosa* was grown overnight from frozen stock. Next morning, the culture was diluted 100-fold by fresh LB medium and grown until OD595 =  ~ 0.6 (~ 6 h). Bacteria were collected by centrifugation and re-suspended in PBS. The dissected corneas were placed on agarose‑gelatin (0.5% each) solid support in a 6-well culture plate containing 800 µl MEM with antibiotics (100 U/ml penicillin, 25 µg/ml spectinomycin), then three horizontal and 3 vertical scratches were made using a sterile 25-gauge needle. 3 × 10^4^ CFU of *P. aeruginosa* ATCC 19660 strain or 0.4 × 10^4^ CFU of PAO1 strain were applied to cornea and incubated for 16–20 h in 37 °C CO_2_ incubator (20% CO_2_). 5 corneas were used for each experimental point. After incubation with *P. aeruginosa*, the corneas were visually inspected for opacity. The corneas which were clear and without signs of infection were considered as non-infected and discarded from further study. 5 µg of S5-PmnH (in 5 μl of PBS) or 5 μl of PBS were applied to infected corneas and incubated for additional 16–20 h in 37 °C CO_2_ incubator.

#### Homogenization and CFU counting

Prior the homogenization the corneas were washed 3 times for 10 min in 50 ml sterile PBS with occasional agitation. Each cornea was chopped in 4 equal parts, trying to get rid of sclera. 2 parts were placed in Precellys 24 tissue homogenizer (Bertin technologies) tubes CKMix50-7 ml and 2 ml PBS was added. Homogenization was performed at following conditions: 6500 rpm 20 s., 5 cycles, 3–5 min breaks on ice between the cycles. The obtained homogenate was transferred into 15 ml Falcon tube and briefly spun to sediment the big debris, then the supernatant recovered into new tube and centrifuged at full speed for 10 min to pellet all bacteria. Bacteria were re-suspended in 200 µl sterile PBS, serially diluted and plated on LB-agar plates.

### Murine keratitis model

#### Animals

The inbred mice of C57BL/6 strain of both sexes in equivalent numbers were used for the research. 2–6 months old, adult female and adult male mice were purchased from the Vilnius University vivarium of laboratory animals. The animals throughout the period of the experiment were given standard chow and drinking water ad libitum. Animals were housed in the individual plastic cages in a 12 h light/dark cycle at 21–23 °C. All regulated procedures on living animals were approved by The Lithuanian Ethics Committee of Biomedical Research (Protocol no. B1-442) and were carried out in accordance with the European Union legislation of OECD (directive 2010/63/EU). The study was carried out in compliance with the ARRIVE guidelines. The experiments were carried out in Biological Research Center of Lithuanian University of Health Sciences.

#### *Pseudomonas aeruginosa* keratitis induction and treatment.

For *P. aeruginosa* infection and keratitis induction the mice were anesthetized by Ketamine and Xylazine 90:9 mg/kg intraperitoneal injection. The cornea of the left eye of each mouse was visualized under a stereoscopic microscope, and three 1 mm scratches were made using a sterile 25-gauge needle. A 10 µl aliquot containing 4 × 10^6^ cells of *P. aeruginosa* ATCC 19660 (cytotoxic strain) or PAO1 ATCC 15692 (invasive strain) was applied to the corneal surface. Depending on the experiment, the treatment was started 30 min or 6 h post infection. 10 µl aliquot containing 0.14 mg of tobramycin or 20 µg of S5-PmnH [both containing 0.5% hydroxypropyl methylcellulose (HPMC)] or PBS containing 0.5% HPMC was applied as one drop of substance to each eye. HPMC is used as artificial tears and was used in order to thicken the tears film and prolong the presence of the applied product on the surface of the cornea. The treatment was continued for five days twice daily. Mice were euthanized by cervical dislocation 1, 3, 5 days post infection, and the eyeballs were collected and homogenized for viable bacteria count.

#### Assessment of clinical score

The eyes were examined and photographed with a dissection microscope equipped with a digital camera at 1, 3 and 5 dpi to monitor the disease progression. At 1, 3 and 5 dpi disease severity was visually graded by using an established corneal damage scale: 0, the pupil was partially or fully covered by clear or slight opacity; + 1, the anterior segment was partially or fully covered by slight opacity; + 2, the pupil was partially or fully covered by dense opacity; + 3, the entire anterior segment was covered by dense opacity; and + 4, corneal perforation^43^.

#### Histopathology

One randomly chosen mice of each study group was used for histopathology experiments. Enucleated eyes were preserved in 10% formaldehyde. Eyes were paraffin-embedded, cut into 3-μm-thick sections, deparaffinised, rehydrated and used for preparation of hematoxylin/eosin-stained samples. All samples were observed with the Eclipse TE2000-U microscope (Nikon, Tokyo, Japan).

### Murine lung colonization and treatment

#### Animals

The studies were performed in Evotec facility under UK Home Office Legislation and Guidelines, with relevant Establishment, Project, and Personal license authorities in place. Local ethical committee approval was in place for this model and all studies were prepared and conducted in keeping with the ARRIVE Guidelines. CD1 male mice were supplied by Charles River (Margate, UK) and were specific pathogen free. Mice were 11 to 15 g on receipt at the facility and were allowed to acclimatize for at least 7 days. Mice were housed in individual ventilated cages always exposing the mice to HEPA filtered air. Mice had free access to food and water and were provided with aspen chip bedding. The room temperature was 22 ± 1 °C, with a relative humidity of 60% and maximum background noise of 56 dB. Mice were exposed to 12-h light/dark cycles.

#### Mice infection and pyocin treatment

*Pseudomonas aeruginosa* ATCC 27853 was cultured on cystine-lactose-electrolyte-deficient (CLED) agar at 37 °C under aerobic conditions for approximately 16–24 h. 20 ml of Mueller Hinton broth was inoculated with a single well isolated colony and cultured overnight at 37 °C with shaking at 300 rpm. The overnight broth was diluted 1:100 in Mueller Hinton broth and 100 ml was cultured in a baffle flask for ~ 6 h at 37 °C with shaking at 300 rpm until the broth OD was ~ 0.6. 20 ml of culture was centrifuged at 2465 g for 10 min and washed in PBS. The pellet was suspended in PBS and the OD_600_ adjusted to 0.67 (~ 2.6 × 10^8^ CFU/ml). The study inoculum was prepared from this by appropriate dilution with PBS. The inoculum concentration was confirmed by quantitative culture on *Pseudomonas* Selective Agar (PSA). The inoculum concentration for the study was 1.47 × 10^7^ CFU/ml (5.87 × 10^5^ CFU/mouse).

Mice were infected under temporary inhaled 3% isoflurane anesthesia by intranasal (IN) instillation with 40 µl of the inoculum suspension split as 20 µl/nostril.

1 h post infection 6 mice were sacrificed in order to evaluate the pre-treatment burden in lungs. Treatments were administered IN once at 1 h post infection (50 µl split to 25 µl/nares). Three treatment groups consisting of six mice each were administered S5-PmnH (2.5, 25 and 250 µg). The fourth group of mice (n = 6) was administered 200 µg Tobramycin (40 mg/ml injection solution, Hospira UK Ltd, diluted 10 times). The study was terminated at 5 h post infection for all animals. The clinical conditions and weights were assessed, and animals were immediately euthanized using an overdose of pentobarbitone. Following confirmation of death, the lungs were excised and homogenized in ice cold sterile phosphate buffered saline using a Precellys bead beater. The homogenates were quantitatively cultured onto PSA agar and incubated at 37 °C for 24 h before colonies were counted.

## Supplementary Information

Supplementary Information.

## Data Availability

All datasets generated or analyzed during this study are included in this published article and supporting information.
