# Supplementary material for: Chimeric bacteriocin S5-PmnH engineered by domain swapping efficiently controls Pseudomonas aeruginosa infection in murine keratitis and lung models
Source: Sci Rep. 2022 Apr 19;12:5865. doi: 10.1038/s41598-022-09865-8 (PMC9018753; doi:10.1038/s41598-022-09865-8)
Supplement: Supplementary file 1 — Supplementary Information. [file 41598_2022_9865_MOESM1_ESM.pdf]

Supporting information

Chimeric bacteriocin S5-PmnH engineered by domain swapping efficiently controls *Pseudomonas aeruginosa* infection in murine keratitis and lung models

Šarūnas Paškevičius, Viktorija Dapkutė, Audrius Misiūnas, Modestas Balzaris, Pia Thommes, Abdul Sattar, Yuri Gleba and Aušra Ražanskienė

Supplementary Text S1. Purification of Chimeric Pyocins.

Frozen leaf tissue was homogenized with chilled mortar and pestle in liquid nitrogen. Prepared powder was mixed with cold extraction buffer (50 mM Tris, 5 mM Sodium acetate, pH 5.0) at a ratio of 1 g of plant material to 5 ml of buffer. The crude extract was incubated at 20-25 °C for 15-20 min. Cell debris were removed by centrifugation at 3220 *g,* at 4 °C for 20 min. Pellets were discarded and the supernatant was filtered through membrane filter (pore size 0.45 µm). Ammonium sulphate was added up to 0.60 M and pH of solution adjusted to 8.0. The formed precipitate was removed by centrifugation at 3220 *g,* at 4 °C for 5 min. The supernatant was taken as total soluble protein and applied for purification in two steps.

At the first purification step the chromatography column was filled with Phenyl sepharose FF resin (GE Healthcare Life Sciences, Uppsala, Sweden) and pre-equilibrated with cold buffer (50 mM Tris, 5 mM Sodium acetate, 0.60 M $(NH_4)_2SO_4$, pH 8.0). Protein solution was loaded to column and the Phenyl sepharose bounded protein fraction was eluted by washing with elution buffer (50 mM Tris, 5 mM Sodium acetate, 0.30 M $(NH_4)_2SO_4$, pH 8.0). Collected protein fraction was loaded into the diafiltrating concentrator (10 kDa) and centrifuged at 3220 *g* until the volume of

protein solution decreased 10 folds. The concentrate was then diluted up to a primary volume with 50 mM Tris and 5 mM Sodium acetate (pH 8.0). The procedure was repeated till conductivity decreased below 5 mS/cm and afterwards the protein solution was subjected to the final purification step using Q sepharose FF resin (GEHealthcare Life Sciences, Uppsala, Sweden). Chromatography media was pre-equilibrated with cold buffer (50 mM Tris, 5 mM Sodium acetate, pH 8.0). Protein solution was loaded to column and Q sepharose unbounded protein was collected in flow-through fraction. Collected protein fraction was loaded into the diafiltrating concentrator (10 kDa) and centrifuged at 3220 *g* until the volume of protein solution decreased 10 folds. The concentrate was diluted up to a primary volume with Phosphate-buffered saline (PBS) buffer. The protein was freeze-dried for long time storage.

Supplementary Text S2. Nucleic and Amino acid sequences of chimeric pyocins. Pyocin S5 part is underlined.

S5-PmnH

ATGTCCAATGATAACGAAGTGCCAGGTTCAATGGTTATAGTTGCTCAAGGTCCAGATGATCAATACGCTTACGAAG
TTCCTCCAATCGATTCTGCTGCTGTTGCTGGAAATATGTTCGGAGATCTTATCCAAAGGGAAATATATCTTCAAAAG
AATATCTATTACCCAGTTAGATCTATCTTCGAGCAAGGAACAAAGGAAAAGAAAGAGATTAATAAGAAAGTTTCA
GATCAAGTTGATGGACTTTTGAAGCAAATTACACAAGGAAAGAGAGAAGCTACTAGGCAAGAGAGAGTTGATGT
TATGTCAGCTGTTCTTCATAAGATGGAATCTGATTTGGAGGGATATAAGAAAACTTTTACAAAGGGTCCTTTTATTG
ATTACGAAAAGCAATCTTCACTTTCAATCTATGAAGCTTGGGTTAAGATTTGGGAGAAAAATTCTTGGGAGGAGA
GAAAGAAATACCCTTTTCAACAACTTGTTAGGGATGAATTGGAGAGAGCTGTTGCTTATTACAAGCAAGATTCTCT
TTCAGAAGCTGTTAAAGTTCTTAGGCAAGAGTTGAATAAGCAAAAGGCTTTGAAGGAAAAGGAGGATCTTTCACA
ATTGGAAAGAGATTACAGGACAAGAAAGGCTAATCTTGAGATGAAGGTTCAATCTGAGTTGGATCAAGCTGGATC
AGCTCTTCCACCTTTGGTTTCTCCAACTCCTGAACAATGGCTTGAGAGGGCTACAAGATTGGTTACTCAAGCTATTG
CTGATAAGAAACAACTTCAAACTACAAATAACACATTGATTAAGAACTCTCCAACTCCTCTTGAAAAGCAAAAAGC
TATCTATAATGGTGAACTTTTGGTTGATGAGATTGCTTCATTGCAAGCTAGGCTTGTTAAATTGAATGCTGAGACTA
CAAGAAGGAGAACAGAAAAGGAACTTACACCAGATGAGAAAGCTGCTTATACTGATGCTGTTTCTTTTGTTTCAAC
AGCTAATGAGCAAATGTTGCAAAAGTACGGTGCTAATCTTTCAAAAGTTGCTCAAGATATGCAAGCTGAGATTGCT

GGAAAGAAAGTTAGGTCTTATGCTGAAGCTATGGCTACTTTTGAGAAGATTTCAGCTAATCCAGCTATGAAACTTA
ATGCTTTGGATACACAAGCTGTTGTTGATGCTCTTAATGCTTTGGATAAGGCTTCTTTCGCTGATAACATCACTAGG
TTGGGAAAGGCTTTTGGTGTTGTTGGAAAAGTTGTTCAAGCTGAAGCTATTAGAGAGAAGACAGTTTCAGGTTTTC
AAACTGGAGATTGGAAACCTTTGATGCTTGAATTGGAGGCTATGGCTGTTGGAACTGGTGCTGGAATTCTTTTGGC
TACATCTATGGCTTTCTTTTTCCCAGTTTTTGCTTCAGCTGCTGCTGGAGTTGTTGTTGTTGCTCTTATGATGGCTGC
TACAGCTGCTTACTTTGATGCTGCTAAGGTTGATGAAATTAATAACCTTATTTTGAATTAA

MSNDNEVPGSMVIVAQGPDDQYAYEVPPIDSAAVAGNMFGDLIQREIYLQKNIYYPVRSIFEQGTKEKKEINKKVSDQ
VDGLLKQITQGKREATRQERVDVMSAVLHKMESDLEGYKKTFTKGPFIDYEKQSSLSIYEAWVKIWEKNSWEERKKYPF
QQLVRDELERAVAYYKQDSLSEAVKVLRQELNKQKALKEKEDLSQLERDYRTRKANLEMKVQSELDQAGSALPPLVSPT
PEQWLERATRLVTQAIADKKQLQTTNNTLIKNSPTPLEKQKAIYNGELLVDEIASLQARLVKLNAETTRRRTEKELTPDEK
AAYTDAVSFVSTANEQMLQKYGANLSKVAQDMQAEIAGKKVRSYAEAMATFEKISANPAMKLNALDTQAVVDALNA
LDKASFADNITRLGKAFGVVGKVVQAEAIREKTVSGFQTGDWKPLMLELEAMAVGTGAGILLATSMAFFFPVFASAAA
GVVVVALMMAATAAYFDAAKVDEINNLILN

S5-Pflu095

ATGTCCAATGATAACGAAGTGCCAGGTTCAATGGTTATAGTTGCTCAAGGTCCAGATGATCAATACGCTTACGAAG
TTCCTCCAATCGATTCTGCTGCTGTTGCTGGAAATATGTTCGGAGATCTTATCCAAAGGGAAATATATCTTCAAAAG
AATATCTATTACCCAGTTAGATCTATCTTCGAGCAAGGAACAAAGGAAAAGAAAGAGATTAATAAGAAAGTTTCA
GATCAAGTTGATGGACTTTTGAAGCAAATTACACAAGGAAAGAGAGAAGCTACTAGGCAAGAGAGAGTTGATGT
TATGTCAGCTGTTCTTCATAAGATGGAATCTGATTTGGAGGGATATAAGAAAACTTTTACAAAGGGTCCTTTTATTG
ATTACGAAAAGCAATCTTCACTTTCAATCTATGAAGCTTGGGTTAAGATTTGGGAGAAAAATTCTTGGGAGGAGA
GAAAGAAATACCCTTTTCAACAACTTGTTAGGGATGAATTGGAGAGAGCTGTTGCTTATTACAAGCAAGATTCTCT
TTCAGAAGCTGTTAAAGTTCTTAGGCAAGAGTTGAATAAGCAAAAGGCTTTGAAGGAAAAGGAGGATCTTTCACA
ATTGGAAAGAGATTACAGGACAAGAAAGGCTAATCTTGAGATGAAGGTTCAATCTGAGTTGGATCAAGCTGGATC
AGCTCTTCCACCTTTGGTTTCTCCAACTCCTGAACAATGGCTTGAGAGGGCTACAAGATTGGTTACTCAAGCTATTG
CTGATAAGAAACAACTTCAAACTACAAATAACACATTGATTAAGAACTCTCCAACTCCTCTTGAAAAGCAAAAAGC
TATCTATAATGGTGAACTTTTGGTTGATGAGATTGCTTCATTGCAAGCTAGGCTTGTTAAATTGAATGCTGAGACTA
CAAGAAGGAGAACAGAAAAGGATAAGGTTAGGACTGAGGTGGTGGACATCGAGGCTGCTATTAAGTTCACCAGC
GACTTCTATGCTGAGGCTGGTGTTAGGTTCGGCGACTCTGCTTCTAAGCTTGCTAGCGATCTTGCTGATAGCGCCC
AGGGTAAGAAAATCAGGTCTGCTGATGAGGCTTTCAAGGCCTTCGACAAGTACAAGAACGAGCTGGACAAGAAG

TTCAGCGTCAAGGATAGAGCTGCTGCCGCTAAGTACATCGACAGCATTGATTACGAGGCTATCGGCAAGGCTGCC
ACCAAGTTCTCTAAAGGTCTTGGTTACGTGGGCCCTGTGATCGATGCTAAGGATAGCATCATCGAGTTCATCAACA
GCATGGAAAGCGGCGACTGGAAGCCATTCTTCCTGAAGCTTGAGTCTATCGCTCTGGGTCTTGCTGCTACTGCTTT
CGTGGGTATTGCCTTCGGTTTCATTGCTACTACCCCTATGGGTATCCTGGCCTTCGCTTTTATTGTGGCTGCTACCG
GTGCTGCTATCGACGATAATTTCGCTGAGAAGCTGAACAAGTTCGTGTCCAGCCTTTAA

<u>MSNDNEVPGSMVIVAQGPDDQYAYEVPPIDSAAVAGNMFGDLIQREIYLQKNIYYPVRSIFEQGTKEKKEINKKVSDQ
VDGLLKQITQGKREATRQERVDVMSAVLHKMESDLEGYKKTFTKGPFIDYEKQSSLSIYEAWVKIWEKNSWEERKKYPF
QQLVRDELERAVAYYKQDSLSEAVKVLRQELNKQKALKEKEDLSQLERDYRTRKANLEMKVQSELDQAGSALPPLVSPT
PEQWLERATRLVTQAIADKKQLQTTNNTLIKNSPTPLEKQKAIYNGELLVDEIASLQARLVKLNAETTRRRTE</u>KDKVRTEV
VDIEAAIKFTSDFYAEAGVRFGDSASKLASDLADSAQGKKIRSADEAFKAFDKYKNELDKKFSVKDRAAAAKYIDSIDYEAI
GKAATKFSKGLGYVGPVIDAKDSIIEFINSMESGDWKPFFLKLESIALGLAATAFVGIAFGFIATTPMGILAFAFIVAATGA
AIDDNFAEKLNKFVSSL

S5-Pflu373

<u>ATGTCCAATGATAACGAAGTGCCAGGTTCAATGGTTATAGTTGCTCAAGGTCCAGATGATCAATACGCTTACGAAG
TTCCTCCAATCGATTCTGCTGCTGTTGCTGGAAATATGTTCGGAGATCTTATCCAAAGGGAAATATATCTTCAAAAG
AATATCTATTACCCAGTTAGATCTATCTTCGAGCAAGGAACAAAGGAAAAGAAAGAGATTAATAAGAAAGTTTCA
GATCAAGTTGATGGACTTTTGAAGCAAATTACACAAGGAAAGAGAGAAGCTACTAGGCAAGAGAGAGTTGATGT
TATGTCAGCTGTTCTTCATAAGATGGAATCTGATTTGGAGGGATATAAGAAAACTTTTACAAAGGGTCCTTTTATTG
ATTACGAAAAGCAATCTTCACTTTCAATCTATGAAGCTTGGGTTAAGATTTGGGAGAAAAATTCTTGGGAGGAGA
GAAAGAAATACCCTTTTCAACAACTTGTTAGGGATGAATTGGAGAGAGCTGTTGCTTATTACAAGCAAGATTCTCT
TTCAGAAGCTGTTAAAGTTCTTAGGCAAGAGTTGAATAAGCAAAAGGCTTTGAAGGAAAAGGAGGATCTTTCACA
ATTGGAAAGAGATTACAGGACAAGAAAGGCTAATCTTGAGATGAAGGTTCAATCTGAGTTGGATCAAGCTGGATC
AGCTCTTCCACCTTTGGTTTCTCCAACTCCTGAACAATGGCTTGAGAGGGCTACAAGATTGGTTACTCAAGCTATTG
CTGATAAGAAACAACTTCAAACTACAAATAACACATTGATTAAGAACTCTCCAACTCCTCTTGAAAAGCAAAAAGC
TATCTATAATGGTGAACTTTTGGTTGATGAGATTGCTTCATTGCAAGCTAGGCTTGTTAAATTGAATGCTGAGACTA
CAAGAAGGAGAACAGAA</u>GCTGAGGAACAGGCTAAGGCTGAGGAAGAGATTAAGGGCGCTATCAAGTTCACCGC
CGACTTCTACAAAGAAATCGGCGAGAAGTACGGCGCTCAGATGACTGCTTCTGCTACTGATCTTGCTGAGACTGCT
AAGGGTAAGACCCTTAGGTCAGCAGAAGAGGCTCTGAAGGCTTTCGATCAGTACAAGGATCACCTGGACAAGAA
GTTCAGCGCTGCTGATAGGGCTGCTATTGTGAACGCTCTGGATTCTCTTGACAGGGCTGAGCTTGCTAAGAACCTG
AACCTGTTCGCTAAAGGCTTCGGCTACACCTCTAAGGCATTCGATGTGTACGATCTGGTGGAAGAGGTGAAGAAA
AGCTACGCTAGCGGCGATTGGAACAACACCGCTCTTAAGGTTGAGACTCTGTTCGCTGGTTCTGCTGCTACCGGTT

TGATTGCTTTCGCTTTCGGTGTGACTGTGTCTACCCCTGTTGGTATTGTGGCTTTCGCTCTGATTATGGCTCTGGTGA
GCGCTTACATCGATGACGCTCATGTGAAGCAGTTCAACGATGCTCTGGATGCTATCCTGCCTTAA

MSNDNEVPGSMVIVAQGPDDQYAYEVPPIDSAAVAGNMFGDLIQREIYLQKNIYYPVRSIFEQGTKEKKEINKKVSDQ
VDGLLKQITQGKREATRQERVDVMSAVLHKMESDLEGYKKTFTKGPFIDYEKQSSLSIYEAWVKIWEKNSWEERKKYPF
QQLVRDELERAVAYYKQDSLSEAVKVLRQELNKQKALKEKEDLSQLERDYRTRKANLEMKVQSELDQAGSALPPLVSPT
PEQWLERATRLVTQAIADKKQLQTTNNTLIKNSPTPLEKQKAIYNGELLVDEIASLQARLVKLNAETTRRRTEAEEQAKAE
EEIKGAIKFTADFYKEIGEKYGAQMTASATDLAETAKGKTLRSAEEALKAFDQYKDHLDKKFSAADRAAIVNALDSLDRA
ELAKNLNLFAKGFGYTSKAFDVYDLVEEVKKSYASGDWNNTALKVETLFAGSAATGLIAFAFGVTVSTPVGIVAFALIMA
LVSAYIDDAHVKQFNDALDAILP

S5-Pflu794

ATGTCCAATGATAACGAAGTGCCAGGTTCAATGGTTATAGTTGCTCAAGGTCCAGATGATCAATACGCTTACGAAG
TTCCTCCAATCGATTCTGCTGCTGTTGCTGGAAATATGTTCGGAGATCTTATCCAAAGGGAAATATATCTTCAAAAG
AATATCTATTACCCAGTTAGATCTATCTTCGAGCAAGGAACAAAGGAAAAGAAAGAGATTAATAAGAAAGTTTCA
GATCAAGTTGATGGACTTTTGAAGCAAATTACACAAGGAAAGAGAGAAGCTACTAGGCAAGAGAGAGTTGATGT
TATGTCAGCTGTTCTTCATAAGATGGAATCTGATTTGGAGGGATATAAGAAAACTTTTACAAAGGGTCCTTTTATTG
ATTACGAAAAGCAATCTTCACTTTCAATCTATGAAGCTTGGGTTAAGATTTGGGAGAAAAATTCTTGGGAGGAGA
GAAAGAAATACCCTTTTCAACAACTTGTTAGGGATGAATTGGAGAGAGCTGTTGCTTATTACAAGCAAGATTCTCT
TTCAGAAGCTGTTAAAGTTCTTAGGCAAGAGTTGAATAAGCAAAAGGCTTTGAAGGAAAAGGAGGATCTTTCACA
ATTGGAAAGAGATTACAGGACAAGAAAGGCTAATCTTGAGATGAAGGTTCAATCTGAGTTGGATCAAGCTGGATC
AGCTCTTCCACCTTTGGTTTCTCCAACTCCTGAACAATGGCTTGAGAGGGCTACAAGATTGGTTACTCAAGCTATTG
CTGATAAGAAACAACTTCAAACTACAAATAACACATTGATTAAGAACTCTCCAACTCCTCTTGAAAAGCAAAAAGC
TATCTATAATGGTGAACTTTTGGTTGATGAGATTGCTTCATTGCAAGCTAGGCTTGTTAAATTGAATGCTGAGACTA
CAAGAAGGAGAACAGAAAAGGCTAGCGACCAGGCTAACAACGTTGACATCGAAGAGGCTATCCTGTTCACCACC
ACCTTCTACGAGAACCTCACTGAGAAGTACGGCGAGAAGGTTAGCGCTGTGGCTAAAGAACTTGCTGAGAGCGCT
AAGGGCAAGACCATGAGGTCATCTAAAGAGGCTCTGCAGACCTTCGAGAAGTACAAGGATACCTACAACGGCCG
GTTCAGGTCTAGGGATAGAAGGGAAGTTGATCGGGCTCTGAAGTCCCTTGACAAAGAGCTGCTGTCTAAGAACCT
CGCCAAGTTCTCTAAGGCTTTCGGCTCCGTGTCTAAGATCGGTGATCTTACCGAGGTGTTCATCGAGTTGGAGAAC
TCTATCAGGACCGGCGATTGGAAGCCTCTTCTGCTTACCCTTGAAGGTATCGGTCTTGGTATGGCTGGAACCTACC
TTGTTGCTGCTGTGTTCGGTATCTCTGCTACTACCCCTCTTGGCATTGTGGTGTTCGCTGTTCTTATGGCTGCTACCA
GCGCTTACATCGATGATGACCTGGTGAAGAAGGTGAACAAGGACCTGTTCGGCTTCTAA

MSNDNEVPGSMVIVAQGPDDQYAYEVPPIDSAAVAGNMFGDLIQREIYLQKNIYYPVRSIFEQGTKEKKEINKKVSDQ
VDGLLKQITQGKREATRQERVDVMSAVLHKMESDLEGYKKTFTKGPFIDYEKQSSLSIYEAWVKIWEKNSWEERKKYPF
QQLVRDELERAVAYYKQDSLSEAVKVLRQELNKQKALKEKEDLSQLERDYRTRKANLEMKVQSELDQAGSALPPLVSPT
PEQWLERATRLVTQAIADKKQLQTTNNTLIKNSPTPLEKQKAIYNGELLVDEIASLQARLVKLNAETTRRRTEKASDQAN
NVDIEEAILFTTTFYENLTEKYGEKVSAVAKELAESAKGKTMRSSKEALQTFEKYKDTYNGRFRSRDRREVDRALKSLDKEL
LSKNLAKFSKAFGSVSKIGDLTEVFIELENSIRTGDWKPLLLTLEGIGLGMAGTYLVAAVFGISATTPLGIVVFAVLMAATS
AYIDDDLVKKVNKDLFGF

S5-Pflu618

ATGTCCAATGATAACGAAGTGCCAGGTTCAATGGTTATAGTTGCTCAAGGTCCAGATGATCAATACGCTTACGAAG
TTCCTCCAATCGATTCTGCTGCTGTTGCTGGAAATATGTTCGGAGATCTTATCCAAAGGGAAATATATCTTCAAAAG
AATATCTATTACCCAGTTAGATCTATCTTCGAGCAAGGAACAAAGGAAAAGAAAGAGATTAATAAGAAAGTTTCA
GATCAAGTTGATGGACTTTTGAAGCAAATTACACAAGGAAAGAGAGAAGCTACTAGGCAAGAGAGAGTTGATGT
TATGTCAGCTGTTCTTCATAAGATGGAATCTGATTTGGAGGGATATAAGAAAACTTTTACAAAGGGTCCTTTTATTG
ATTACGAAAAGCAATCTTCACTTTCAATCTATGAAGCTTGGGTTAAGATTTGGGAGAAAAATTCTTGGGAGGAGA
GAAAGAAATACCCTTTTCAACAACTTGTTAGGGATGAATTGGAGAGAGCTGTTGCTTATTACAAGCAAGATTCTCT
TTCAGAAGCTGTTAAAGTTCTTAGGCAAGAGTTGAATAAGCAAAAGGCTTTGAAGGAAAAGGAGGATCTTTCACA
ATTGGAAAGAGATTACAGGACAAGAAAGGCTAATCTTGAGATGAAGGTTCAATCTGAGTTGGATCAAGCTGGATC
AGCTCTTCCACCTTTGGTTTCTCCAACTCCTGAACAATGGCTTGAGAGGGCTACAAGATTGGTTACTCAAGCTATTG
CTGATAAGAAACAACTTCAAACTACAAATAACACATTGATTAAGAACTCTCCAACTCCTCTTGAAAAGCAAAAAGC
TATCTATAATGGTGAACTTTTGGTTGATGAGATTGCTTCATTGCAAGCTAGGCTTGTTAAATTGAATGCTGAGACTA
CAAGAAGGAGAACAGAAGCTGAAGCTGAGGCTAAGAGAATTGATGATTACAAAAGGGCTGTTGCTTTCGTTGCT
GATGCTAATAAGTACATCTTGGAAAAGTACGGTGCTAATCTTCATCAAGTTGTTATGGATTTGCAAAAGGATATCT
CTGGAAAGAAAATTAGATCATACGCTGAAGCTATGCAAACTTTTGAGGCTGTTAGAACAAATCCAAATGCTAGGCT
TTCTCCTCAAGATACTAGGGCTGTTGTTGATGCTTTGAATGCTCTTGATAAGGCTACATACATGGATTCTGTTAATA
AGTTGGCTAAAGGATTTGGTGTTACTGGAAAGATTGTTCAAGCTCATTCAGTTATTGAAAAGACAGTTATTGGTTT
TAGAGATGGAAATTGGAAACCACTTATTTTGGAACTTGAGTCAATTGCTTTGGGAGCTGGTGCTGGAGCTGCTGTT
GCTACACTTTTGGCTGTTTTCTCTCCTGGATTTGCTGCTTCAGCTATTGGTATTGTTGCTGTTGGAGTTGCTATTGCT
ACTATTGCTTCTCTTTTGAACGCTGATAACGTTGAGAAGATTAATGTTTTTATTTCAGATCATTTGGAAACAGCTCTT
AAAGAGCAAAGGTAA

MSNDNEVPGSMVIVAQGPDDQYAYEVPPIDSAAVAGNMFGDLIQREIYLQKNIYYPVRSIFEQGTKEKKEINKKVSDQ
VDGLLKQITQGKREATRQERVDVMSAVLHKMESDLEGYKKTFTKGPFIDYEKQSSLSIYEAWVKIWEKNSWEERKKYPF

QQLVRDELERAVAYYKQDSLSEAVKVLRQELNKQKALKEKEDLSQLERDYRTRKANLEMKVQSELDQAGSALPPLVSPT
PEQWLERATRLVTQAIADKKQLQTTNNTLIKNSPTPLEKQKAIYNGELLVDEIASLQARLVKLNAETTRRRTEAEAEAKRI
DDYKRAVAFVADANKYILEKYGANLHQVVMDLQKDISGKKIRSYAEAMQTFEAVRTNPNARLSPQDTRAVVDALNAL
DKATYMDSVNKLAKGFGVTGKIVQAHSVIEKTVIGFRDGNWKPLILELESIALGAGAGAAVATLLAVFSPGFAASAIGIV
AVGVAIATIASLLNADNVEKINVFISDHLETALKEQR

S5-Ppu259

ATGTCCAATGATAACGAAGTGCCAGGTTCAATGGTTATAGTTGCTCAAGGTCCAGATGATCAATACGCTTACGAAG
TTCCTCCAATCGATTCTGCTGCTGTTGCTGGAAATATGTTCGGAGATCTTATCCAAAGGGAAATATATCTTCAAAAG
AATATCTATTACCCAGTTAGATCTATCTTCGAGCAAGGAACAAAGGAAAAGAAAGAGATTAATAAGAAAGTTTCA
GATCAAGTTGATGGACTTTTGAAGCAAATTACACAAGGAAAGAGAGAAGCTACTAGGCAAGAGAGAGTTGATGT
TATGTCAGCTGTTCTTCATAAGATGGAATCTGATTTGGAGGGATATAAGAAAACTTTTACAAAGGGTCCTTTTATTG
ATTACGAAAAGCAATCTTCACTTTCAATCTATGAAGCTTGGGTTAAGATTTGGGAGAAAAATTCTTGGGAGGAGA
GAAAGAAATACCCTTTTCAACAACTTGTTAGGGATGAATTGGAGAGAGCTGTTGCTTATTACAAGCAAGATTCTCT
TTCAGAAGCTGTTAAAGTTCTTAGGCAAGAGTTGAATAAGCAAAAGGCTTTGAAGGAAAAGGAGGATCTTTCACA
ATTGGAAAGAGATTACAGGACAAGAAAGGCTAATCTTGAGATGAAGGTTCAATCTGAGTTGGATCAAGCTGGATC
AGCTCTTCCACCTTTGGTTTCTCCAACTCCTGAACAATGGCTTGAGAGGGCTACAAGATTGGTTACTCAAGCTATTG
CTGATAAGAAACAACTTCAAACTACAAATAACACATTGATTAAGAACTCTCCAACTCCTCTTGAAAAGCAAAAAGC
TATCTATAATGGTGAACTTTTGGTTGATGAGATTGCTTCATTGCAAGCTAGGCTTGTTAAATTGAATGCTGAGACTA
CAAGAAGGAGAACAGAAGCAGAGGCTGAGGCTAAGAGGATCGATGATTACAAGCGGGCTGTTGCTTACGTGGCC
GATGCTAACAAGTTCATCCTCGAGAAGTACGGTGCCAAGCTTCATCAGGTGGTGATGGATCTGCAGAAGGACGTT
AGCGGCAAGAAAATCCGGTCTTACAACGAGGCTCTTAGGACCTTTGAGCAGGTCAGGACTAATCCTAACGCTAGG
TTGTCTCCTCAGGACACTAGGGCTGTTGTGGATGCTCTTAACGCTCTGGACAAGGCCACCTACATGGATTCTGTGA
ACAGGCTGGCTAAAGGTTTCGGTGTGACCGGTAAGATTGTGCAGGCTCATTCTGTGGTCGAGAAGGCTATTATCG
GTTTCCAGGATGGCAACTGGAAGCCTCTTTTGCTCGAGTTCGAGTCTATTGCTGCTGGTGCTGGTGCAGGTTTGCT
TGTGGCTCTTATTGCTCCTCCTGTGCTGGCTGCTTTTTCTTTCCCACCTGTGATTGCTGTGGTGGCTACTGGTCTTTT
GGTTGCTGGTGTTGCTGCTCTGCTTGACGCTAAGACTGTCGAGAAGATCAACGACACCATCTTCACCCTGGTTGAG
ACTACTCCTGCTCACTAA

MSNDNEVPGSMVIVAQGPDDQYAYEVPPIDSAAVAGNMFGDLIQREIYLQKNIYYPVRSIFEQGTKEKKEINKKVSDQ
VDGLLKQITQGKREATRQERVDVMSAVLHKMESDLEGYKKTFTKGPFIDYEKQSSLSIYEAWVKIWEKNSWEERKKYPF
QQLVRDELERAVAYYKQDSLSEAVKVLRQELNKQKALKEKEDLSQLERDYRTRKANLEMKVQSELDQAGSALPPLVSPT
PEQWLERATRLVTQAIADKKQLQTTNNTLIKNSPTPLEKQKAIYNGELLVDEIASLQARLVKLNAETTRRRTEAEAEAKRI

DDYKRAVAYVADANKFILEKYGAKLHQVVMDLQKDVSGKKIRSYNEALRTFEQVRTNPNARLSPQDTRAVVDALNALD
KATYMDSVNRLAKGFGVTGKIVQAHSVVEKAIIGFQDGNWKPLLLEFESIAAGAGAGLLVALIAPPVLAAFSFPPVIAVV
ATGLLVAGVAALLDAKTVEKINDTIFTLVETTPAH

Supplementary Table S1. ***P. aeruginosa strains*** **used in the study.**

P. aeruginosa **strain**	**Isolated from**	**Source**
Boston 41501 (ATCC 27853)	Blood culture	LGC ATCC
PAO1 (ATCC 15692, DSM 22644)	Infected wound	Leibniz Institut DSMZ-Deutsche Sammlung von Mikroorganismen und Zellkulturen GmbH
PA14 (DSM19882)		Leibniz Institut DSMZ-Deutsche Sammlung von Mikroorganismen und Zellkulturen GmbH
Bu002 LMG24892	Infected wound	The BCCM/LMG bacteria collection
A19 LMG25088	Infected wound	The BCCM/LMG bacteria collection
Pr335 LMG24969	Hospital environment	The BCCM/LMG bacteria collection
EY76	Infected eye	A. Vitkauskienė LSMU
BL77	Blood	A. Vitkauskienė LSMU
UR78	Urinary tract infection	A. Vitkauskienė LSMU
BR79	Bronchus	A. Vitkauskienė LSMU
BI80	Biopsy	A. Vitkauskienė LSMU
HP1	Hospital pneumonia	A. Vitkauskienė LSMU
HP6	Hospital pneumonia	A. Vitkauskienė LSMU
HP7	Hospital pneumonia	A. Vitkauskienė LSMU
HP40	Hospital pneumonia	A. Vitkauskienė LSMU
HP41	Hospital pneumonia	A. Vitkauskienė LSMU
HP52	Hospital pneumonia	A. Vitkauskienė LSMU
HP75	Hospital pneumonia	A. Vitkauskienė LSMU
PA-103	Sputum of patient	ATCC 29260
12-35708	Cystic fibrosis	A. Vitkauskienė LSMU
13-18499	Cystic fibrosis	A. Vitkauskienė LSMU
12-29165	Cystic fibrosis	A. Vitkauskienė LSMU
180 (ATCC 19660)	Human septicemia	LGC ATCC

NCTC 13437		Public Health England Culture collections
NCTC 13921	Human throat	Public Health England Culture collections

Supplementary Table S2. Primers used to amplify receptor-binding- and translocation domain of S5 and pore-forming domains of PmnH, Pflu095, Pflu794, Pflu618 and Pput259. In *italics* - BsaI site, underlined – overlapping sequence obtained after amplification, **bold** – sequence complementary to template (synthetic gene with plant-optimized codons).

**Primer**	**Sequence**
S5 fwd	AAA***GGTCTC*****ACATGTCCAATGATAAC**
S5 rev	AAA*GGTCTC*A**TTCTGTTCTCCTTCTTGTAGTCT**
PmnH fwd	AAA*GGTCTC*AAGAA**AAGGAACTTACACCAGATGAGAA**
PmnH rev	AAA*GGTCTC*A**AAGCTTAATTCAAAATAAG**
Pflu095 fwd	AAA*GGTCTC*AAGAA**AAGGATAAGGTTAGGACTGAGG**
Pflu095 rev	AAA*GGTCTC*A**AAGCTTAAAGGCTGG**
Pflu794 fwd	AAA*GGTCTC*AAGAA**AAGGCTAGCGACCAGGCTAAC**
Pflu794 rev	AAA*GGTCTC*A**AAGCTTAGAAGCCGA**
Pflu618 fwd	AAA*GGTCTC*AAGAA**GCTGAAGCTGAGGCTAAGAGA**
Pflu618 rev	AAA*GGTCTC*A**AAGCTTACCTTTGCTC**
Pput259 fwd	AAA*GGTCTC*AAGAA**GCAGAGGCTGAGGCTAAGAG**
Pput259 rev	AAA*GGTCTC*A**AAGCTTAGTGAGCAG**

Supplementary Figure S1.

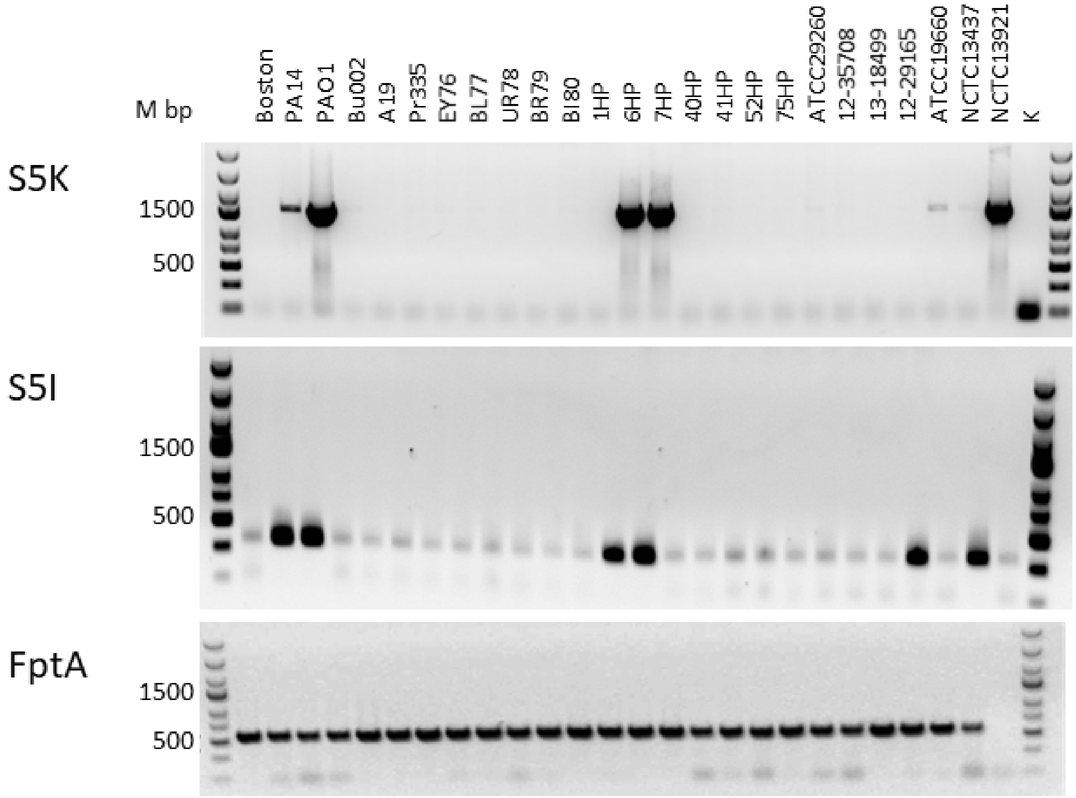

Suppl. Fig S1. PCR amplification of S5 killing and immunity genes and fptA from genomic DNA of 25 *P. aeruginosa* isolates. Sequence – specific primers were designed to amplify 1500 bp fragment of S5K, 330 bp fragment of S5I and 556 bp fragment of fptA. FptA is amplified in all tested strains, S5K and S5I was amplified in PA14, PAO1, HP6, HP7, ATCC 19660 and NCTC 13921 strains. K – negative control.

Supplementary Figure S2.

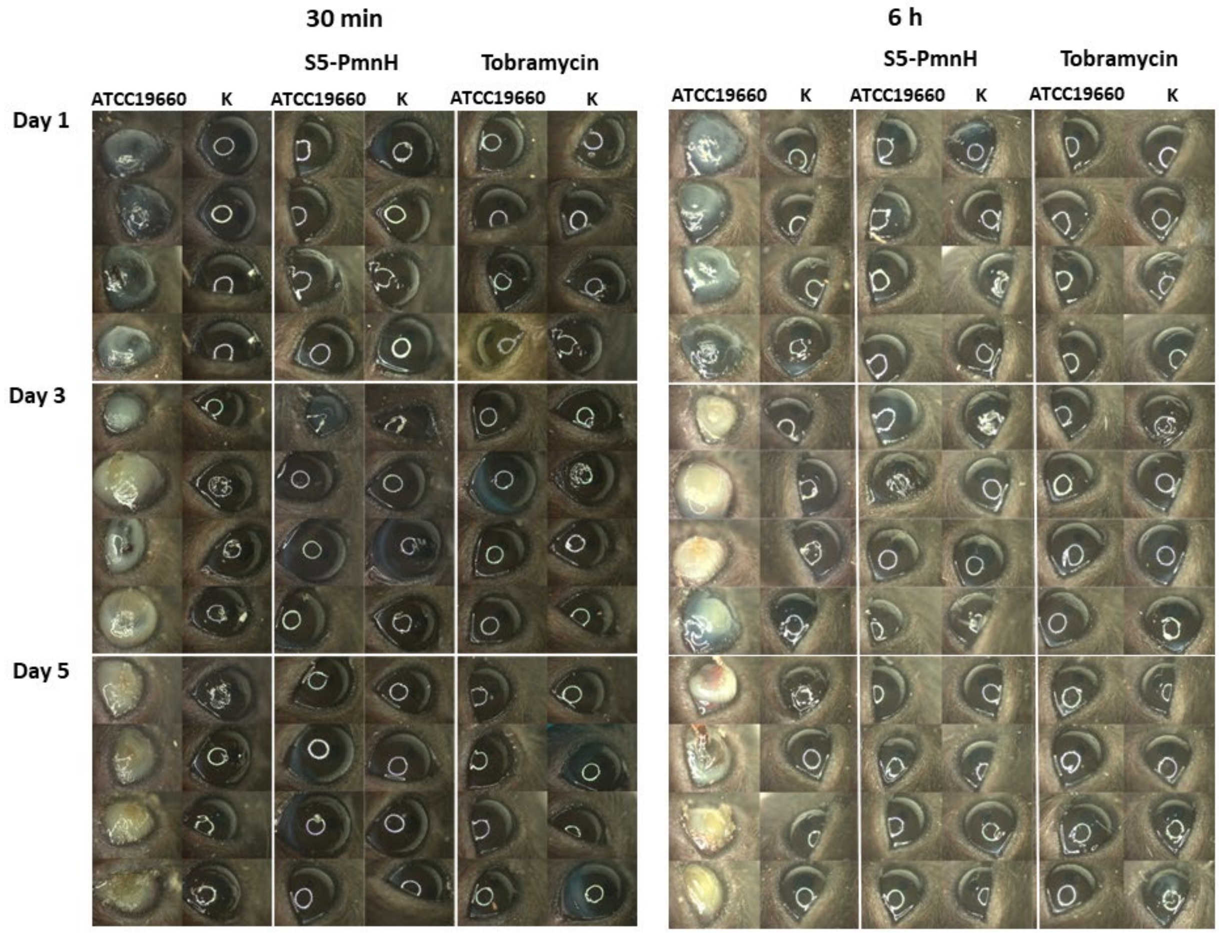

Suppl. Fig. S2. Slit lamp microscopy images of mice eyes infected by cytotoxic strain ATCC 19660 and treated by S5-PmnH or tobramycin. A. The eyes of all animals used for experiment (both infected and healthy control eyes) were photographed with a dissection microscope equipped with a digital camera at 1, 3 and 5 dpi.

Supplementary Figure S3.

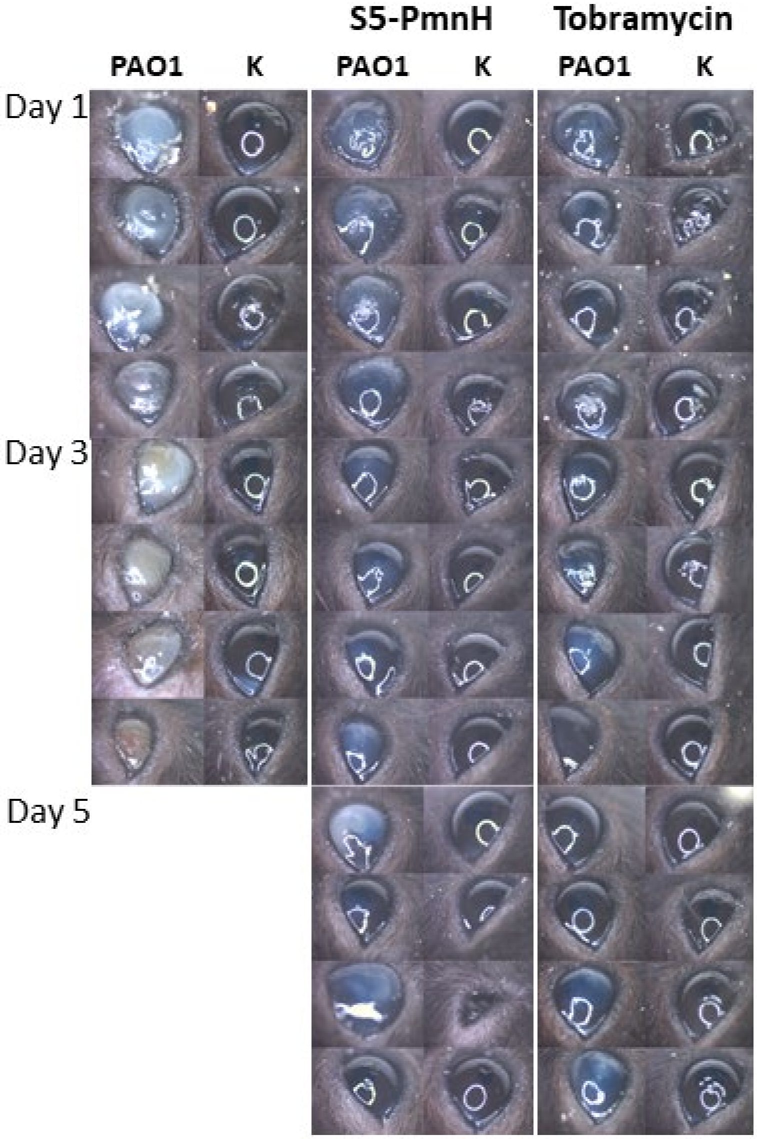

Suppl. Fig. S2. Slit lamp microscopy images of mice eyes infected by invasive strain PAO1 and treated by S5-PmnH or tobramycin. A. The eyes of all animals used for experiment (both infected and healthy control eyes) were photographed with a dissection microscope equipped with a digital camera at 1, 3 and 5 dpi.
